# Comparative analysis of nanomechanical resonators: sensitivity, response time, and practical considerations in photothermal sensing

**DOI:** 10.1038/s41378-025-00879-6

**Published:** 2025-02-18

**Authors:** Kostas Kanellopulos, Friedrich Ladinig, Stefan Emminger, Paolo Martini, Robert G. West, Silvan Schmid

**Affiliations:** Institute of Sensor and Actuator Systems, TU Wien, Gusshausstrasse 27-29, Vienna, 1040 Austria

**Keywords:** NEMS, Sensors

## Abstract

Nanomechanical photothermal sensing has significantly advanced single-molecule/particle microscopy and spectroscopy, and infrared detection. In this approach, the nanomechanical resonator detects shifts in resonant frequency due to photothermal heating. However, the relationship between photothermal sensitivity, response time, and resonator design has not been fully explored. This paper compares three resonator types - strings, drumheads, and trampolines - to explore this relationship. Through theoretical modeling, experimental validation, and finite element method simulations, we find that strings offer the highest sensitivity (with a noise equivalent power of 280 fW/Hz^1/2^ for strings made of silicon nitride), while drumheads exhibit the fastest thermal response. The study reveals that photothermal sensitivity correlates with the average temperature rise and not the peak temperature. Finally, the impact of photothermal back-action is discussed, which can be a major source of frequency instability. This work clarifies the performance differences and limits among resonator designs and guides the development of advanced nanomechanical photothermal sensors, benefiting a wide range of applications.

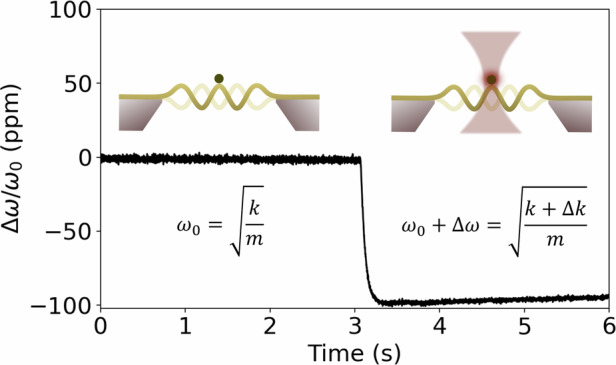

## Introduction

In nanomechanical photothermal sensing, the mechanical resonator detects heat generated from various processes, including electromagnetic radiation absorption^1–7^ and non-radiative energy transfer from minute samples^8–15^, single molecules^16^, single nanoparticles^17–23^, two-dimensional (2D) materials^24,25^, and thin films^26,27^. In this approach, a tensile stressed nanomechanical resonator functions as the sensing element for the detection of energy exchange with the environment via resonance frequency shifts. As the resonator absorbs heat, its temperature rises, which decreases the tensile stress, leading to a corresponding frequency detuning. In other words, the resonator operates as a mechanical thermometer, offering flexibility in terms of optimal sensor design and material choice^28^. In particular, when transduced optically, the mechanical photothermal resonator can guarantee operation limited fundamentally by thermomechanical and photothermal back-action noise, in contrast to, e.g., thermoelectric sensors and detectors, which are plagued by electronic noise^29^.

**Fig. 1:**
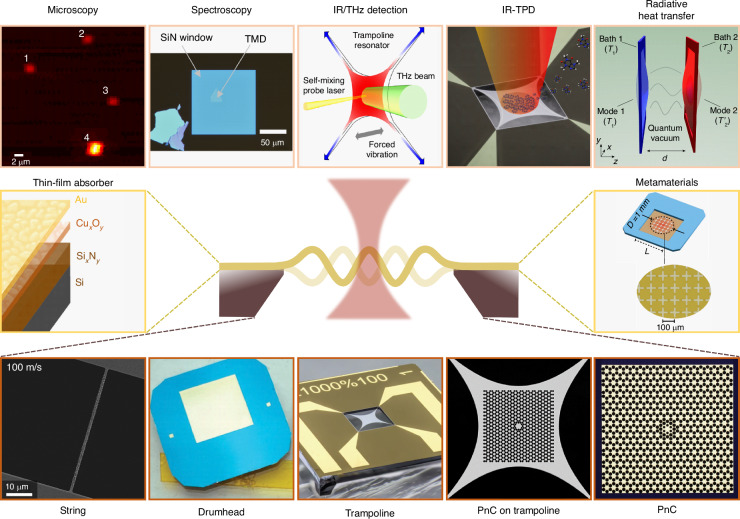
Nanomechanical photothermal sensing. To date, the photothermal effect in nanomechanical resonators has been explored in different fields of application (top row): molecular microscopy (reproduced and cropped from ref. ^16^--Copyright author(s) 2018, licensed under CC BY-NC-ND 4.0) and spectroscopy (reproduced and cropped from ref. ^25^--Copyright 2023 under a CC BY 4.0 license), IR/THz detection (reproduced and cropped from ref. ^4^--Copyright 2022 under a CC BY 4.0 license), IR-temperature programmed desorption (IR-TPD, reproduced and cropped from ref. ^15^--Copyright 2023 under a CC BY 4.0 license), and radiative heat transfer mechanisms (adapted with permission from ref. ^37^--Copyright 2019 by Springer Nature), among others. Within this wealth of studies, different resonator designs have been used (bottom row): strings (reproduced and cropped from ref. ^11^--Copyright 2016 under a CC BY-NC-ND 4.0 license), drumheads (adapted with permission from ref. ^33^--Copyright 2023 by AIP Publishing), trampolines (reproduced and cropped from ref. ^5^--Copyright 2023 under a CC BY 4.0 license), phononic crystal (PnC) geometries on trampolines (reproduced and cropped from ref. ^61^--Copyright 2020 under a CC BY 4.0 license), and PnC alone (adapted with permission from ref. ^64^--Copyright 2017 by Springer Nature). Depending on the application, addition of a further layer on top of the sensing area is also possible. Two examples are (central row): thin-film absorber (reproduced and cropped from ref. ^65^--Copyright 2020 under a CC BY 4.0 license), and metamaterials (reproduced and rearranged from ref. ^7^--Copyright 2024 under a CC BY 4.0 license)

Over the last decade, nanomechanical photothermal sensing has emerged as a powerful detection approach due to its high temperature sensitivity and versatility. This is evident in the rapidly expanding areas of application, as outlined in Fig. 1. To date, this technique has shown competitive performances in the field of molecular microscopy and spectroscopy, operating in a wide range of the electromagnetic spectrum, from the visible^16,17,20–22^, near-infrared (IR)^23–25^, to mid-IR^8,9,11,12,15,26,27^. In addition, the field is making important steps toward bridging the terahertz (THz) gap with resonant micro- and nanomechanical thermal detectors, offering a unique approach for room-temperature operation^1–5,7,30–33^. Light-sound interaction in nanoresonators has been also successfully employed for enthalpy measurements^34^, and detection of near-field heat radiation transfer^35,36^, as well as phonon heat transfer through vacuum fluctuations^37^.

So far, a variety of mechanical photothermal sensors has been employed for all this wealth of results, driven by different experimental requisites. For instance, silicon nitride string resonators have been extensively explored for photothermal sensing, in particular for microscopy and spectroscopy applications^8–11,13,14,17,18,20,38^. Their small cross-sectional area, together with the mechanical and thermal properties of SiN, make them sensitive to temperature changes of the orders of few *μ*K^38^. With their high surface area, drumheads have been another platform of choice for nanomechanical photothermal spectroscopy of single nano-absorber^16,22,23^, 2D materials^24,25^ and thin films^26,27^, as well as for detection of IR/THz radiation^3,5,7,35,36^. As it will be shown here, this advantage comes at the expense of a reduced thermal sensitivity. Recently, trampoline resonators have also been employed in a variety of works in photothermal sensing^4,5,39–41^, due to their improved thermal insulation compared to drumheads, leading to competitive sensitivities with respect to the strings. However, no comprehensive modeling and comparison of different resonator designs has been performed with respect to their sensitivity, response time, and practicality.

**Fig. 2:**
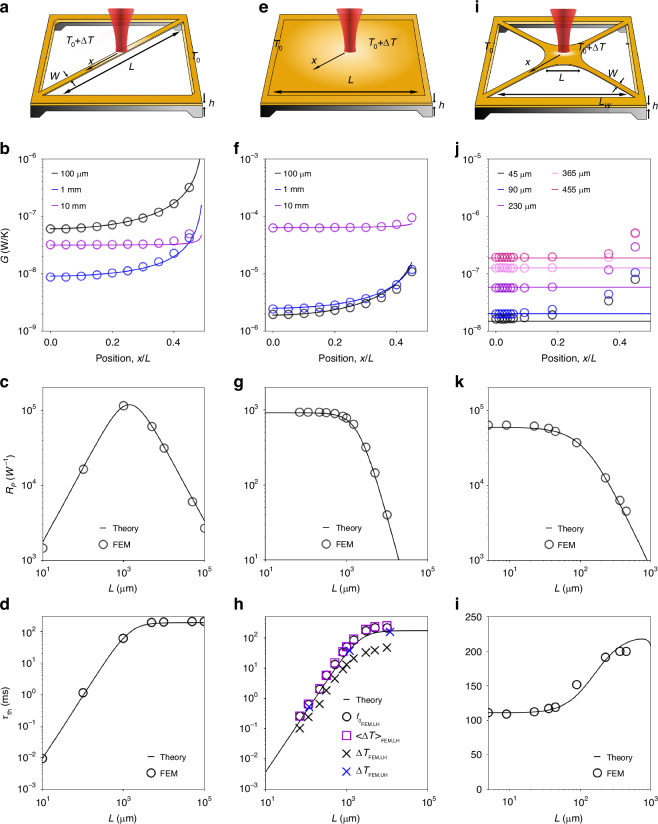
FEM validation of MTF. **a** Schematics of a string resonator illuminated by a light source (red) at the center. At steady-state, a temperature difference Δ*T* from the frame temperature *T*_0_, will arise upon photothermal heating. **b** String’s thermal conductance *G* as a function of the point-like heat source relative position *x*/*L*, for three different lengths (0.1, 1, and 10 mm). Circles: FEM results of *G* in the mean temperature framework (MTF). Solid curve: MTF theoretical calculation (15). **c** Comparison between FEM results (circles) and model (solid curve) for the relative power responsivity (3) as a function of the string length. **d** Comparison between FEM results (circles) and model (solid curve) for the thermal time constant (4) as a function of the string length. **e** Schematics of a drumhead resonator. For the comparison with the FEM, an equivalent circular geometry is used to reduce the problem complexity. **f** Circular drumhead’s MTF thermal conductance for 0.1, 1, and 10 mm side length. **g** Relative power responsivity comparison for drumheads. **h** Thermal time constant comparison for drumheads. Solid curve: theory (4). Blue crosses: response time of the FEM peak temperature Δ*T*_*FEM*_ for uniform heating (UH). Black crosses: response time of Δ*T*_*FEM*_ for local heating (LH). Purple squares: response time of the surface mean temperature $\left\langle \Delta {T}_{FEM}\right\rangle$ for LH. Black circles: response time of the resonance frequency ${f}_{{0}_{FEM}}$ for LH. **i** Schematics of a trampoline resonator. **j** Trampolines' MTF thermal conductance for a frame window side length of 1.1 mm and five different central pad side lengths. The model (solid curve) accounts here for a heat source impinging only in the central pad. **k** Relative power responsivity comparison for trampolines. **l** Thermal time constant comparison for trampolines. Model and FEM used parameters for all the designs: *ρ* = 3000 kg/m^3^, *c*_*p*_ = 700 J/(kg K), *κ* = 2.7 W/(mK), *E* = 250 GPa, *σ*_0_ = 200 MPa, *ν* = 0.23, *α*_th_ = 1.23 ppm/K, *ϵ*_rad_ = 0.05, *α* = 0.5%, *w* = 5 *μ*m, *h* = 50 nm^30,61^. For all the FEM simulations, a Gaussian beam of waist *w*_0_ = 1 *μ*m has been used

This study establishes a comprehensive theoretical framework aimed at assessing the photothermal sensing performance of nanomechanical resonators, with a focus on noise equivalent power and response time. The analytical models illuminate, in particular, the interplay between sensor responsivity and frequency stability^7^. Models for the individual noise components of the frequency stability are derived, including additive phase noise, temperature fluctuation noise, and photothermal back-action noise. The models herein are rigorously validated through comparison with experimental data and finite element method (FEM) simulations across varied nanomechanical silicon nitride resonator designs, namely strings, square drumheads, and trampolines, as schematically depicted in Fig. 2a, e, i.

## Theory

Photothermal sensors measure the power absorbed by the mechanical resonator, *P*. This is the fraction of irradiated or otherwise introduced power *P*_0_ that is converted into heat

1 $$P=\alpha {P}_{0}$$

with the absorber- and wavelength-dependent heat conversion factor *α* (0 ≤ *α* ≤ 1). In this context, the figure of merit is the noise-equivalent-power (NEP) with units [${\rm{W}}/\sqrt{{\rm{Hz}}}$], which for nanomechanical resonators is defined as^42^

2 $$\,\text{NEP}\,=\frac{\sqrt{{S}_{y}(\omega )}}{{{\mathcal{R}}}_{{\rm{P}}}(\omega )},$$

with the one-sided noise power spectral density (PSD) *S*_*y*_(*ω*) of the fractional frequency *y*, with units [1/Hz], the relative power responsivity ${{\mathcal{R}}}_{{\rm{P}}}(\omega )$, with units [1/W], where *ω* denotes the angular speed [rad Hz].

The NEP indicates the minimum detectable power per unit bandwidth, assuming a signal-to-noise ratio of unity. In other words, it quantifies the sensor’s power sensitivity.

The relative responsivity is defined as the fractional shift of the resonator eigenfrequency *ω*_0_ per absorbed power *P* and is given by^42^

3 $${{\mathcal{R}}}_{{\rm{P}}}(\omega )=\frac{\partial {\omega }_{0}}{\partial {\rm{P}}}\frac{1}{{\omega }_{0}}\left\vert {H}_{{\rm{th}}}(\omega )\right\vert =\,\frac{{{\mathcal{R}}}_{{\rm{T}}}}{G}\left\vert {H}_{{\rm{th}}}(\omega )\right\vert ,$$

where ${{\mathcal{R}}}_{{\rm{T}}}$ denotes the relative responsivity to temperature *T* with units [1/K], *G* is the resonator’s thermal conductance [W/K], and ${H}_{{\rm{th}}}(\omega )={(1+i\omega {\tau }_{{\rm{th}}})}^{-1}$ a low-pass filter transfer function accounting for the resonators’ thermal response time^43^

4 $${\tau }_{{\rm{th}}}=\,\frac{C}{G},$$

with *C* denoting the resonators’ heat capacity [J/K].

### Temperature responsivity

According to Eq. (3), nanomechanical photothermal sensors are, in essence, temperature sensors. The temperature responsivity is defined as

5 $${{\mathcal{R}}}_{{\rm{T}}}=\frac{\partial {\omega }_{0}}{\partial T}\frac{1}{{\omega }_{0}({T}_{0})},$$

with *T*_0_ being the thermal bath temperature. The eigenfrequency of the resonators considered in this work is a function of their temperature-dependent tensile stress *σ*(*T*)

6 $${\omega }_{0}\propto \sqrt{\sigma (T)},$$

while the effect of bending stiffness is neglected (for *σ*_0_ ≥ 1 MPa). In the following, it is assumed that only the mechanical resonator is heated to a temperature *T*, while keeping its frame at a fixed temperature *T*_0_ < *T*. The resulting temperature increase Δ*T* = *T* − *T*_0_ is responsible for the frequency shift experienced by the resonator.

#### Strings

In a string resonator with an intrinsic uniaxial tensile stress *σ*_0_ and Young’s modulus *E*, a mean temperature increase $\left\langle \Delta T\right\rangle$ induces a thermal strain along the resonator’s length *L*, resulting in a stress^42^

7 $$\sigma (T)=\,{\sigma }_{0}-\,E\,{\alpha }_{{\rm{th}}}\,\left\langle \Delta T\right\rangle ,$$

with *α*_th_ being the material’s linear coefficient of thermal expansion. For small temperature changes, the temperature responsivity (5) together with (6) and (7) approximately is given by

8 $${{\mathcal{R}}}_{{\rm{T}}}=-\frac{{\alpha }_{{\rm{th}}}}{2}\frac{E}{{\sigma }_{0}}.$$

The factor *E*/*σ*_0_ is called the photothermal enhancement factor and is a unique feature of resonators under tensile stress. For nanomechanical silicon nitride resonators, the photothermal enhancement factor can reach values between 10^2^ and 10^8^. It is worth noting here that, for 10kPa ≤ *σ*_0_ ≤ 1 MPa, the thermal stress (second addend in Eq. (7)) can be of the same order of magnitude of *σ*_0_, making the temperature responsivity (8) nonlinear (as observed in ref. ^16^). For *σ*_0_ < 10 kPa, the resonator behaves as a beam, with a reduced *R*_T_ = (*α*_th_ + *α*_E_)/2 (*α*_E_ being the Young’s modulus softening coefficient). The same discussion is valid for drumheads.

#### Drumheads

For very thin (*h* ≪ *L*) homogeneous isotropic drumheads, the assumption of thin shell holds, and the thermal stress is given by^44^

9 $$\sigma (T)=\,{\sigma }_{0}\,-\,\frac{{\alpha }_{{\rm{th}}}\,E}{1\,-\,\nu }\left\langle \Delta T\right\rangle ,$$

with *ν* being the resonator’s Poisson’s ratio. Hence, the relative temperature responsivity is by (see Eq. (S23) for the full expression and related discussion)

10 $${{\mathcal{R}}}_{{\rm{T}}}=\,-\,\frac{{\alpha }_{{\rm{th}}}}{2\,(1\,-\,\nu )}\frac{E}{{\sigma }_{0}},$$

with the factor (1 − *ν*) accounting for the thermal expansion along the two in-plane directions (for a detailed discussion about the dependence of ${{\mathcal{R}}}_{T}$ on the heat localization in drumheads, see SI Section S3).

#### Trampolines

The trampolines, as the one depicted in Fig. 2i, exhibit a thermal response similar to strings. For a central pad of area *L*^2^ and thickness *h*, anchored to the frame via four tethers of length *L*_t_, width *w*, and thickness *h*, its effective spring constant for the fundamental resonance mode can be expressed as that of string of length *L*_t_ (see SI Section S4)

11 $${k}_{{\rm{eff}}}(T)=\frac{{\pi }^{2}}{2}\frac{wh}{{L}_{{\rm{t}}}}(1-\nu ){\sigma }_{0}\left[1-\frac{{\alpha }_{{\rm{th}}}E}{{\sigma }_{0}}\left\langle \Delta T\right\rangle \right],$$

with the factor (1 − *ν*) accounting here for the strain release along the directions perpendicular to the tether length. From the resonance frequency ${\omega }_{0}(T)\propto \sqrt{{k}_{{\rm{eff}}}}$, it is easy to observe that the temperature responsivity is equal to

12 $${{\mathcal{R}}}_{{\rm{T}}}=-\frac{{\alpha }_{{\rm{th}}}}{2}\frac{E}{{\sigma }_{0}},$$

underlining that the thermal expansion at the tethers is the main responsible for the temperature response (see SI Section S4).

### Thermal conductance

Besides the temperature responsivity, the power responsivity (3) also depends on the thermal conductance. The following thermal analysis is carried out based on the mean temperature framework (MTF) in the steady state that we introduce here. The model is derived first assuming a point-like heat source. The case of an evenly spread heat source is discussed in the end of each subsection.

A resonator of thermal mass *C* absorbs a power *P* and dissipates it to the environment through its conductance *G*, resulting in a mean temperature rise $\left\langle \Delta T\right\rangle$

13 $$\left\langle \Delta T\right\rangle =\frac{P}{G}.$$

In the MTF, all the resonator thermal properties are defined with respect to $\left\langle \Delta T\right\rangle$, as this temperature dictates the photothermal response of a nanomechanical resonator under tensile stress, rather than the local temperature variations Δ*T* (for a detailed discussion, see Fig. 2h and SI Section S3).

For an isotropic resonator, *C* is given in the MTF by

14 $$C=\,{c}_{{\rm{p}}}\,\rho \,V,$$

where *c*_*p*_, *ρ*, and *V* are the specific heat capacity at constant pressure, mass density, and volume of the resonator, respectively.

As the resonator operates in a vacuum environment (see Materials and methods), only thermal conduction *G*_cond_ and radiation *G*_rad_ contribute to the heat transfer^45^. In the MTF, the thermal conductance *G* is given by

15 $$G={G}_{{\rm{rad}}}+{G}_{{\rm{cond}}}=4{A}_{{\rm{rad}}}{\epsilon }_{{\rm{rad}}}{\sigma }_{{\rm{SB}}}{T}_{0}^{3}+\frac{{s}_{{\rm{f}}}({\bf{r}},L,{w}_{0})}{\beta ({\bf{r}},L,{w}_{0})}\kappa ,$$

where *A*_rad_, *ϵ*_rad_, *κ*, and *σ*_SB_ are the resonator’s radiating surface, its emissivity, thermal conductivity, and the Stefan-Boltzmann constant, respectively. For the thermal conduction term *G*_cond_, a shape factor *s*_f_ is introduced to account for the design geometry via the resonator characteristic length *L*, the heat source position vector **r**, and the heating radius *w*_0_^45^. In this way, the dependence of *G* on the size of the probing heat source, as well as on its position with respect to the resonator, e.g., concentric or eccentric to it, are taken into account within this formalism. The product *s*_f_(**r**, *L*, *w*_0_) ⋅ *κ* is the thermal conduction with respect to the localized temperature field Δ*T*. The factor *β*(**r**, *L*, *w*_0_) denotes the ratio between mean and peak temperature $\beta =\left\langle \Delta T\right\rangle /\Delta T$, ensuring the correct description of *G*_cond_ in the MTF. In contrast, *G*_rad_ proves to be independent of the localization conditions of the laser and its position (see SI Fig. S9c), and the resonator’s full area is considered.

#### Strings

A string (Fig. 2a) of length *L*, width *w*, and thickness *h* occupies a volume *V* = *hwL*, and, assuming *h* ≪ *w*, radiates with an area *A*_rad_ ≈ 2*wL*. The factor of 2 accounts for the front and back surface radiation. This allows the direct evaluation of the thermal capacitance *C* and radiative conductance *G*_rad_. For *G*_cond_ instead, shape *s*_f_ and *β* factors have to be calculated. In this regard, it should be noted that the heating source can be in any position *x* along the string length, with the generated heat flowing along two paths of length *x* and *L* − *x*^28,43^. For a point-like heat source (*w*_0_ → 0), *s*_f_ and *β* are given by

16 $${s}_{{\rm{f}}}(x)=\,\frac{4\,w\,h}{L}\,\frac{1}{1\,-\,{\left(\frac{2x}{L}\right)}^{2}}\,,\,\,\,\text{and}\,$$

17 $$\beta (x)=\,\frac{1}{2}.$$

Eq. (17) is valid as long as the temperature profile is linear, for which $\left\langle \Delta T\right\rangle =\Delta T/2$ (see SI Section S3).

**Fig. 3:**
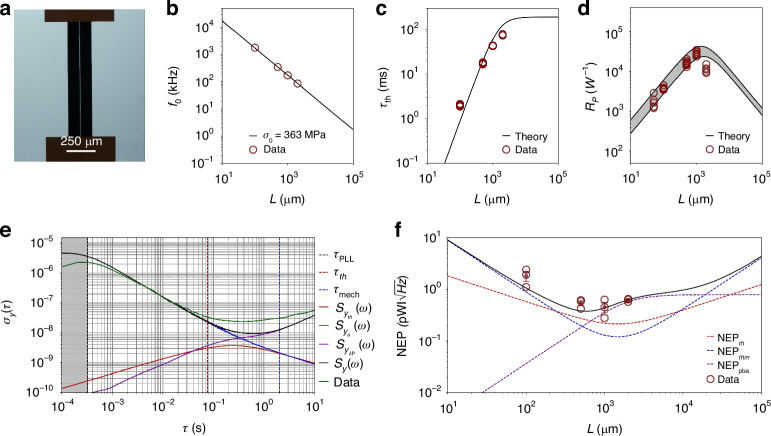
String design. **a** Optical micrograph of a string resonator. **b** Resonance frequency of 56 nm thick, 5 *μ*m wide string resonators of different lengths. From the measurements, a stress of 363 MPa is extracted. **c** Comparison between theoretical (black solid curve) and measured (dark red circles) thermal time constant *τ*_th_ for the same set of strings. **d** Comparison between theoretical (solid curves) and measured (dark red circles) relative power responsivity. The error bar indicates the uncertainties in *κ* (2.7 − 4 W/(m K)), *E* (200 − 300 GPa)^16^. For these structures, *α*_th_ = 1 ppm/K has been measured following ref. ^38^. **e** Allan deviation measured for a 2 mm long string (green solid curve), driven at the onset of nonlinearity ${z}_{{{\rm{r}}}_{{\rm{c}}}}$, with low-pass filter bandwidth ${f}_{{\rm{demod}}}=2.5\,{\rm{kHz}}$, PLL bandwidth *f*_PLL_ = 500 Hz and optical input power *P*_0_ = 6 *μ*W. The comparison with the theoretical model is also shown (black solid curve), together with the single contributions (see main text). The grey region includes all the processes faster than the PID controller (*τ* < *τ*_PLL_), which has a low-pass behavior, filtering all the fast processes out. **f** Comparison between the theoretical (black solid curve) and experimentally extracted (black circles) NEP for strings. The theoretical curve is composed of two different noise contributions: temperature (red dashed curve) and thermomechanical (blue dashed curve) fluctuations-induced fractional frequency noise. For each string’s length, three different resonators were characterized in terms of NEP. Average and standard mean error for the data points are also shown for each length

Figure 2b shows the overall thermal conductance *G*(15) as a function of the relative localized heating position for three different strings. The MTF model (solid curves) closely aligns with the FEM simulations (circles), where the conductance has been extracted as ${G}_{{\rm{FEM}}}=P/\left\langle \Delta {T}_{{\rm{FEM}}}\right\rangle$. For strings measuring 0.1 mm and 1 mm in length (black and blue curves, respectively), *G* strongly depends on the heat source position, increasing as the latter approaches the frame, due to the enhanced thermal conduction. This effect is less pronounced for the 10 mm long string, where radiative heat transfer dominates. It is worth noting that the 1 mm long string shows the best thermal insulation, followed by the 10 mm long and 0.1 mm resonators, consistent with the theoretical and experimentally determined power responsivity ${{\mathcal{R}}}_{{\rm{P}}}$ (see Fig. 2c & 3d).

For the case that the heating point source is located in the string center (*x* = 0), *G*_cond_ can be expressed as

18 $${G}_{{\rm{cond}}}=\frac{{s}_{{\rm{f}}}(x=0)}{\beta (x=0)}\,\kappa =\,\frac{8\,h\,w}{L}\,\kappa .$$

The MTF predicts a factor of 2 higher than what is reported in ref. ^42^, as $\left\langle \Delta T\right\rangle$ is considered instead of the peak temperature, and is consistent with ref. ^46^.

Figure 2c compares the theoretical power responsivity (3) to the FEM simulations, showing excellent agreement. For short lengths (*L* < 1 mm), ${{\mathcal{R}}}_{{\rm{P}}}$ increases linearly with *L*, until it reaches a maximum (*L* ≈ 1 mm). In this region, the string is mainly coupled to the thermal bath via thermal conduction (*G* = *G*_cond_). As the distance between the impinging and anchoring points increases, so does the power responsivity. For *L* > 1mm, the string enters the radiation limited regime (*G* = *G*_rad_), resulting in a linear reduction of ${{\mathcal{R}}}_{{\rm{P}}}$, due to the increasingly larger emitting surface area *A*_rad_ ∝ *L*. This comparison proves the validity of the responsivity model.

The thermal conductance (15) also depends on the spot size of the probing laser. FEM simulations for a uniformly heated string shows that thermal insulation is 1.5 × less than under localized heating conditions. Thus, localizing the heat source at the string center will lead to a 1.5 × higher power response (see SI Section S3).

Figure 2d displays the comparison between FEM (circles) and theoretical response time (4). Short strings are dominated by conductive heat transfer, with *τ*_th_ being a linear function of *L*. Conversely, long strings are dominated by radiative heat transfer and show a time constant independent of *L*, as both the thermal capacitance *C* and the conductance *G* = *G*_rad_ grow linearly with *L*. As can be observed, the model accurately predicts the string’s time constant. Notably, there is a trade-off between power responsivity and thermal time constant: for *L* ≤ 1 mm higher responsivity corresponds to a slower thermal time response.

It is worth noting here that, for probing wavelengths *λ* ≥ *w*, scattering phenomena have to be accounted for to extract the correct absorbed power *P*.

#### Drumheads

A square drumhead resonator of side length *L* and thickness *h* (Fig. 2e) has a volume *V* = *hL*^2^ and a radiating surface *A*_rad_ = 2*L*^2^. Given *h* ≪ *L*, no thermal gradient is present along the thickness, and the eccentric shell model applies for the correct description of *G*_cond_^45^, with the heat being dissipated isotropically to the frame. For simplicity, the model focuses on a circular drumhead of effective diameter $D=2L/\sqrt{\pi }$^12,31^. For this geometry, the shape *s*_f_ and *β* factors are given by (for the derivation, see SI Section S3)

19 $${s}_{{\rm{f}}}({\bf{r}},D,{w}_{0})=\,\frac{4\,\pi \,h}{2\,{\cosh }^{-1}\left[\frac{{D}^{2}\,+\,{(2{w}_{0})}^{2}\,-\,4\,{r}^{2}}{2\,D\,(2\,{w}_{0})}\right]+1},$$

20 $$\beta ({\bf{r}},D,{w}_{0})=\,\frac{1-\frac{1}{2}{\left(\frac{2{w}_{0}}{D}\right)}^{2}}{1-2\,{\rm{\ln }}\left(\frac{2\,{w}_{0}}{D}\right)}\left[1-{\left(\frac{r}{D}\right)}^{2}\right].$$

Figure 2f shows the overall conductance *G* as a function of a localized heat source position, for three different circular membranes (*L* = 0.1, 1, and 10 mm). The MTF model (solid curves) closely aligns with the FEM simulations for circular drumheads (circles). The two smaller drumheads (*L* < 1 mm, black and blue curves), primarily coupled to the environment via conduction, exhibit similar values. Conversely, the larger drumhead in the radiative heat transfer regime has a constant and worse thermal conductance.

For a focused heat source at the drumhead center (*r* = 0, *w*_0_ → 0), the conductance becomes

21 $${G}_{{\rm{cond}}}=\frac{{s}_{{\rm{f}}}(r=0)}{\beta (r=0)}\,\kappa =\,4\,\pi \,h\,\kappa ,$$

recovering the same result as ref. ^12^. Even in the case of a localized heat source, thermal conduction in drumheads is independent of the side length *L*, contrary to what happens in strings (18).

Figure 2g shows the comparison between the theoretical (black solid curve) and FEM power responsivity (black circle), showing excellent agreement. Small drumheads (*L* < 1 mm) shows a responsivity independent of *L*, being *G* = *G*_cond_ exclusively a function of the material thermal conductivity *κ* and the resonator’s thickness *h*(21). Large drumheads (*L* > 1 mm) enter the radiative regime, and the responsivity drops down due to the increased surface area. This comparison confirms the validity of the theoretical responsivity model for drumhead resonators.

Drumheads show a different dependence on heat localization compared to strings. FEM simulations for a concentric Gaussian beam of varying waist *w*_0_ have shown that the power responsivity ${{\mathcal{R}}}_{P}({w}_{0}=L/2)\approx {{\mathcal{R}}}_{P}({w}_{0}\ll L/2)/2$, i.e. for a uniform heating condition (see SI Section S3). As a simple rule here, a point-like heat source offers a 2 × improved photothermal responsivity compared to uniformly distributed heating.

Figure 2h compares the theoretical and FEM modeling of the thermal time constant. The study considers uniform (UH) and local heating (LH) conditions. The theoretical predictions (black curve) closely align with the scenario of uniform light illumination (blue crosses), with *τ*_th_ derived from the temporal evolution of the resonator’s maximum temperature Δ*T*. Notably, the thermal equilibrium is reached faster in the case of local heating (black crosses). For the same scenario, *τ*_th_ has been additionally estimated through a transient study of the resonance frequency (black circles), revealing a stronger agreement with the theory. Monitoring the mean temperature $\left\langle \Delta T\right\rangle$ (dark purple squares) further supports this result: the two sets of FEM perfectly overlap, indicating that the resonance frequency is governed by the resultant mean temperature distribution even in the presence of a local heating source. Opposite to what has been seen for strings, the most responsive drumheads show the fastest time response.

#### Trampolines

A trampoline (Fig. 2i) occupies a volume $V=h\left({L}^{2}+4w{L}_{{\rm{t}}}\right)$ and radiates through its central pad and tethers with an area ${A}_{{\rm{rad}}}=2\left({L}^{2}+4w{L}_{{\rm{t}}}\right)$. The 2D heat conduction problem simplifies here to a 1D scenario, as in strings. Indeed, heat generated in any position on the central pad conductively dissipates through the tethers. Since the heat flow is constricted by the tethers, the resonator can be modeled as a cross-string. According to (16), the resulting shape and *β* factors are given by

22 $${s}_{{\rm{f}}}(x)=\,2\,\frac{4\,w\,h}{2\,{L}_{{\rm{t}}}}\frac{1}{1-{\left(\frac{x}{{L}_{{\rm{t}}}}\right)}^{2}},\,\,\,\text{and}\,$$

23 $$\beta ({\bf{r}},L,{w}_{0})=\,1.$$

The factor of 2 in Eq. (22) accounts for the two crossing strings, while Eq. (23) is defined only with respect to the central pad, being the core sensing area.

Figure 2j shows the FEM computed values for *G* for five trampolines of different central areas *L*^2^ (circles), together with the MTF predictions (solid curves). As the heat source moves from the center to the frame along a tether, the thermal conduction *G*_cond_ increases. Moreover, both *G*_cond_ and *G*_rad_ rise for increasing area—the former due to shorter tethers, the latter due to a larger surface. For a tightly focused beam at the center, the thermal conductance is

24 $${G}_{{\rm{cond}}}=4\,\frac{h\,w\,\kappa }{{L}_{{\rm{t}}}},$$

recovering the results of a cross-string resonator of different tether lengths.

Figure 2k displays the theoretical and FEM simulated power responsivity as a function of the central side length *L*. The model aligns closely with FEM simulations: resonators with small areas (*L*^2^ < 100^2^ *μ*m^2^) show an almost constant ${{\mathcal{R}}}_{{\rm{P}}}$; for larger trampolines (*L*^2^ > 100^2^ *μ*m^2^), it decreases linearly as the pad area grows. The trend is similar to the drumhead case (Fig. 2g). The difference in orders of magnitude compared to the drumheads relates to the improved thermal insulation (see Fig. 2f*,* j). As the window size is kept fixed, the growth of the central area corresponds to a reduction in tethers’ length. For *L* < 100 *μ*m, long tethers provide high thermal insulation, with ${{\mathcal{R}}}_{{\rm{P}}}$ converging to the cross-string case. As *L*^2^ approaches ${L}_{{\rm{w}}}^{2}$, thermal radiation, as well as conduction increases due to the tethers shortening, with ${{\mathcal{R}}}_{{\rm{P}}}$ approaching the drumhead performances. This comparison shows the validity of the thermal model employed so far.

As only the central pad is here the sensing area, uniform heating would result in an almost identical mean temperature rise $\left\langle \Delta T\right\rangle$ for this geometry, leading to no reduction of the power responsivity ${{\mathcal{R}}}_{P}({w}_{0}\to 0)\approx {{\mathcal{R}}}_{P}({w}_{0}=L/2)$ (see SI Section S3).

Figure 2l shows the thermal time constant comparison between the model (solid curve) and the FEM simulations (circles), showing excellent agreement. For *L* < 50 *μ*m, the trampoline behaves as a string. For 50 *μ*m < *L* < 230 *μ*m, the resonator thermal capacitance grows faster than the conductance, increasing the overall response time. For *L* > 230 *μ*m, *τ*_th_ reaches a plateau, to drop down for increasingly larger central pads. This is explained by the increase in conduction and radiation: the former, due to the shorter tether length; the latter, due to a bigger central area *L*^2^. The interplay between thermal mass and conductance is the same one observed between the effective mass and the stiffness for the resonance frequency, as shown in Fig. 6b (see also SI Section S4).

As for drumheads, the most responsive trampolines exhibit the fastest time response.

A summary of the expressions of ${{\mathcal{R}}}_{{\rm{T}}}$ and *G* for the calculation of the power responsivity (3) is displayed in Table 1, for point-like source and even illumination.

**Table 1:** Expressions for the relative temperature responsivity ${{\mathcal{R}}}_{{\rm{T}}}$ and thermal conductance *G* for the three designs

	${{\mathcal{R}}}_{{\rm{T}}}$ [1/K]	LH: *G* [W/K]	UH: *G* [W/K]
String	$-\frac{{\alpha }_{{\rm{th}}}}{2}\frac{E}{{\sigma }_{0}}$	$8\frac{wh}{L}\kappa +8wL{\epsilon }_{{\rm{rad}}}{\sigma }_{{\rm{SB}}}{T}_{0}^{3}$	$12\frac{wh}{L}\kappa +8wL{\epsilon }_{{\rm{rad}}}{\sigma }_{{\rm{SB}}}{T}_{0}^{3}$
Drumhead	$-\frac{{\alpha }_{{\rm{th}}}}{2(1-\nu )}\frac{E}{{\sigma }_{0}}$	$4\pi h\kappa +4{L}^{2}{\epsilon }_{{\rm{rad}}}{\sigma }_{{\rm{SB}}}{T}_{0}^{3}$	$8\pi h\kappa +8{L}^{2}{\epsilon }_{{\rm{rad}}}{\sigma }_{{\rm{SB}}}{T}_{0}^{3}$
Trampoline	$-\frac{{\alpha }_{{\rm{th}}}}{2}\frac{E}{{\sigma }_{0}}$	$8\frac{wh}{{L}_{{\rm{t}}}}\kappa +4(8w{L}_{{\rm{t}}}+2{L}^{2}){\epsilon }_{{\rm{rad}}}{\sigma }_{{\rm{SB}}}{T}_{0}^{3}$	$8\frac{wh}{{L}_{{\rm{t}}}}\kappa +4(8w{L}_{{\rm{t}}}+2{L}^{2}){\epsilon }_{{\rm{rad}}}{\sigma }_{{\rm{SB}}}{T}_{0}^{3}$

The two quantities are used to calculate the relative power responsivity (3), for localized (LH) and uniform (UH) heating. For the drumheads see also SI Section S3

### Frequency stability

High photothermal sensitivity (2) requires also low fractional frequency noise, as it defines the smallest resonance frequency shift that can be resolved. In nanomechanical photothermal sensing, the most relevant noise sources are: i) additive phase noise *θ*, the sum of thermomechanical *θ*_thm_ and detection noise ${\theta }_{\det }$, with a frequency fluctuations PSD ${S}_{{y}_{\theta }}(\omega )={S}_{{y}_{{\theta }_{{\rm{thm}}}}}(\omega )+{S}_{{y}_{{\theta }_{\det }}}(\omega )$^47,48^; ii) temperature fluctuation frequency noise, with PSD ${S}_{{y}_{{\rm{th}}}}(\omega )$^33^; and iii) photothermal back-action frequency noise, with PSD ${S}_{{y}_{\delta {\rm{P}}}}(\omega )$^49^

25 $${S}_{y}(\omega )={S}_{{y}_{\theta }}(\omega )+{S}_{{y}_{{\rm{th}}}}(\omega )+{S}_{{y}_{\delta {\rm{P}}}}(\omega ).$$

The experimental frequency fluctuations for all the designs are characterized by the Allan deviation *σ*_y_(*τ*) (AD)^50^

26 $${\sigma }_{{\rm{y}}}(\tau )=\sqrt{\frac{1}{2(N-1)}\mathop{\sum }\limits_{i=1}^{N}{({y}_{i+1,\tau }-{y}_{i,\tau })}^{2}},$$

with *y*_*i*_ being the *i*th sample of the fraction frequency *y*(*t*) averaged over a time *τ*

27 $${y}_{i,\tau }=\frac{1}{\tau }\mathop{\int}\nolimits_{(i-1)\tau }^{i\tau }y(t)dt.$$

The theoretical calculations of the AD are based on the analytical expression^47–49^

28 $${\sigma }_{{\rm{y}}}(\tau )=\sqrt{\frac{1}{2\pi }\frac{8}{{\tau }^{2}}\mathop{\int}\nolimits_{0}^{\infty }\frac{{\sin }^{4}\left(\frac{\omega \tau }{2}\right)}{{\omega }^{2}}{S}_{{\rm{y}}}(\omega )d\omega }.$$

For the specific case of white frequency noise, i.e., ${S}_{{\rm{y}}}(\omega )={\rm{constant}}$, Eq. (28) reduces to

29 $${\sigma }_{{\rm{y}}}(\tau )=\sqrt{\frac{{S}_{{\rm{y}}}(0)}{2\tau }}.$$

#### Additive phase noise

Additive phase noise originates from the conversion into phase noise of thermomechanical *z*_thm_ and detection ${z}_{\det }$ amplitude noise, with respective PSDs ${S}_{{z}_{{\rm{thm}}}}(\omega )$ and ${S}_{{z}_{\det }}(\omega )$^48^. In the assumption of detection white noise, this contribution can be expressed with respect to the thermomechanical noise peak as^42^

30 $${S}_{{z}_{\det }}(\omega )={{\mathcal{K}}}_{{\rm{d}}}^{2}{S}_{{z}_{{\rm{thm}}}}({\omega }_{0})={{\mathcal{K}}}_{{\rm{d}}}^{2}\left[\frac{4{k}_{{\rm{B}}}TQ}{{m}_{{\rm{eff}}}{\omega }_{0}^{3}}\right],$$

with ${{\mathcal{K}}}_{{\rm{d}}} < 1$ for transduction systems able to resolve the thermomechanical noise. *Q* = *ω*_0_*τ*_mech_/2 denotes the quality factor of the resonator, with *τ*_mech_ being the resonator’s mechanical time constant. Assuming that the resonator is made to oscillate at an amplitude *z*_r_ by means of a closed-loop frequency tracking scheme, the resulting fractional frequency noise power spectral density (PSD) is^42^

31 $${S}_{{y}_{\theta }}(\omega )=\frac{1}{2{Q}^{2}}\frac{{S}_{{z}_{{\rm{thm}}}}}{{z}_{{\rm{r}}}^{2}}\left[| {H}_{{\theta }_{{\rm{thm}}}}({\rm{i}}\omega ){| }^{2}+{{\mathcal{K}}}_{{\rm{d}}}^{2}| {H}_{{\theta }_{\det }}({\rm{i}}\omega ){| }^{2}\right].$$

${H}_{{\theta }_{{\rm{thm}}}}({\rm{i}}\omega )$ and ${H}_{{\theta }_{\det }}({\rm{i}}\omega )$ are the loop-specific transfer functions for the thermomechanical and detection phase noise. The transfer functions for an open loop, phase-locked loop, and self-sustaining oscillator are the same to a good approximation^48^. As an example, for a self-sustaining oscillator (SSO) scheme, as used in this work, the transfer functions are^48^

32 $$\begin{array}{rlr}&{H}_{{\theta }_{{\rm{thm}}}}^{{\rm{SSO}}}({\rm{i}}\omega )={H}_{{\rm{L}}}({\rm{i}}\omega ),&\\ &{H}_{{\theta }_{\det }}^{{\rm{SSO}}}({\rm{i}}\omega )=\frac{{H}_{{\rm{L}}}({\rm{i}}\omega )}{{H}_{{\rm{mech}}}({\rm{i}}\omega )}.\end{array}$$

*H*_mech_(i*ω*) and *H*_L_(i*ω*) are the low-pass filter transfer functions of the resonator and system filter, respectively

33 $$\begin{array}{rlr}&{H}_{{\rm{mech}}}({\rm{i}}\omega )=\frac{1}{1+{\rm{i}}\omega {\tau }_{{\rm{mech}}}},&\\ &{H}_{{\rm{L}}}({\rm{i}}\omega )=\frac{1}{1+{\rm{i}}\omega {\tau }_{{\rm{L}}}}.\end{array}$$

with *τ*_mech_ = 2*Q*/*ω*_0_ and the filter time constant *τ*_L_ ≡ 1/(2*πf*_L_), being *f*_L_ the filter bandwidth.

Additive phase noise (31) can be mitigated by actuating the resonator at the onset of nonlinearity ${z}_{{{\rm{r}}}_{{\rm{c}}}}$,

34 $${z}_{{{\rm{r}}}_{{\rm{c}}}}=\,\sqrt{\frac{8}{3\,\sqrt{3}}}\frac{1}{\sqrt{Q}}\sqrt{\frac{{m}_{{\rm{eff}}}\,{\omega }_{0}^{2}}{{\alpha }_{{\rm{Duff}}}}},$$

with *α*_Duff_ denoting the effective Duffing term^42^. For ${z}_{{\rm{r}}} > {z}_{{{\rm{r}}}_{{\rm{c}}}}$, additional phase noise of nonlinear origin could enter the system, worsening the resonator frequency stability at the integration times of interest in this study^51^.

#### Temperature fluctuation frequency noise

Thermal fluctuation fractional frequency noise can also be assumed to be white^43^. For a lumped-element model, ${S}_{{y}_{{\rm{th}}}}(\omega )$ is given by^43,52^

35 $${S}_{{y}_{{\rm{th}}}}(\omega )=\,\frac{4\,{k}_{B}\,{T}^{2}}{{G}_{{\rm{eff}}}}{{\mathcal{R}}}_{{\rm{T}}}^{2}{\left\vert \frac{1}{1+i\omega {\tau }_{{{\rm{th}}}_{{\rm{eff}}}}}\right\vert }^{2}{\left\vert {H}_{{\theta }_{{\rm{thm}}}}({\rm{i}}\omega )\right\vert }^{2}.$$

Here, *G*_eff_ and ${\tau }_{{{\rm{th}}}_{{\rm{eff}}}}$ represent an effective thermal conductance and time constant, accounting for the temperature fluctuations originating from the fluctuating radiant power exchange between resonator and surroundings^53^. Since this can occur at any position onto the detector, the radiant power sources are modelled as point-like heaters. Hence, *G*_eff_ is derived from the integration of the conductance *G* over all possible positions of a point-like heat noise source. Since radiation is heat source position-independent in MTF, only the integration of *G*_cond_ is required. From *G*_eff_, ${\tau }_{{{\rm{th}}}_{{\rm{eff}}}}=C/{G}_{{\rm{eff}}}$ can be evaluated.

In a resonator, thermal noise can enter the system at any point along its length *L*. For a string, integrating Eq. (16) along *L* gives the effective conductance

36 $$\begin{array}{rc}&{G}_{{\rm{eff}}}={\left(\frac{1}{\kappa }\frac{1}{L}\mathop{\int}\nolimits_{0}^{L}\frac{1}{{s}_{{\rm{f}}}(x)}\text{d}x\right)}^{-1}+8wL{\epsilon }_{{\rm{rad}}}{\sigma }_{{\rm{SB}}}{T}_{0}^{3}\\ &=\frac{6\kappa wh}{L}+8wL{\epsilon }_{{\rm{rad}}}{\sigma }_{{\rm{SB}}}{T}_{0}^{3}.\end{array}$$

Eq. (36) results in a higher conductance than (18), as the averaging includes noise sources closer to the clamping points, where *G*_cond_ increases exponentially (see Fig. 2b).

For a circular drumhead, the integration is performed over its whole area, leading to all the possible noise source positions gives

37 $$\begin{array}{lll}{G}_{{\rm{eff}}}={\left(\frac{1}{\pi {(\frac{D}{2})}^{2}\kappa }\mathop{\int}\nolimits_{0}^{2\pi }\mathop{\int}\nolimits_{0}^{D/2-{w}_{0}}\frac{1}{{s}_{{\rm{f}}}(r,\theta ,D,{w}_{0})}r\text{d}r\text{d}\theta \right)}^{-1}\\\qquad +\,8{L}^{2}{\epsilon }_{{\rm{rad}}}{\sigma }_{{\rm{SB}}}{T}_{0}^{3}\\\qquad \simeq 4\pi h\kappa +8{L}^{2}{\epsilon }_{{\rm{rad}}}{\sigma }_{{\rm{SB}}}{T}_{0}^{3}.\end{array}$$

Here, the greatest noise contribution is in the central region, resulting in *G*_eff_ ≃ *G*(**r** = 0), since *G*_cond_ is less influenced by the position of the noisy heating source than in strings (see the ratio between temperature peaks for a heat at the center and closest to the frame in Fig. (S3) and (S5b)).

For a trampoline, the integration is performed over its central pad and along its four tethers, resulting in

38 $$\begin{array}{rlr}&{G}_{{\rm{eff}}}={\left(\frac{1}{\kappa }\frac{1}{\sqrt{2}{L}_{{\rm{w}}}}\mathop{\int}\nolimits_{0}^{\sqrt{2}{L}_{w}}\frac{1}{{s}_{{\rm{f}}}(x)}\text{d}x\right)}^{-1}&\\ &+4(8w{L}_{{\rm{t}}}+2{L}^{2}){\epsilon }_{{\rm{rad}}}{\sigma }_{{\rm{SB}}}{T}_{0}^{3}\\ &=\frac{6\,\sqrt{2}\,\kappa \,w\,h}{{L}_{{\rm{w}}}}+4(8w{L}_{{\rm{t}}}+2{L}^{2}){\epsilon }_{{\rm{rad}}}{\sigma }_{{\rm{SB}}}{T}_{0}^{3}.\end{array}$$

with *L*_w_ denoting the window side length. While trampolines dissipate $\sqrt{2}\times$ more than strings via conduction, the central pad will make this geometry extremely sensitive to temperature fluctuations ($w < L\le \sqrt{4{L}_{{\rm{t}}}w}$, see also the theoretical curves in Fig. 6f).

#### Photothermal back-action frequency noise

**Fig. 4:**
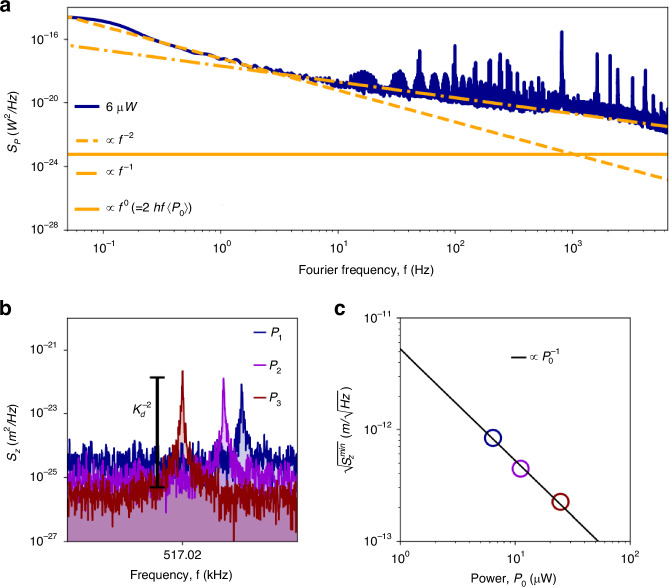
Laser intensity fluctuations. **a** Characterization of the intensity fluctuations for an average power $\left\langle {P}_{0}\right\rangle =6\,\mu {\rm{W}}$ (smoothed with a Gaussian filter). The *f*^−2^ and *f*^−1^ noise contributions are shown (dashed orange and dashed-dotted orange lines, respectively). **b** Power spectral density of the thermomechanical noise for a drumhead resonator for different interferometer laser powers. **c** Corresponding measured displacement sensitivity $\sqrt{{S}_{z}^{min}}$, in units [${\rm{m}}/\sqrt{{\rm{Hz}}}$]. It improves linearly with the laser power, with the effect of simultaneously introducing higher thermomechanical and laser power instability-induced frequency noise

Photothermal back-action frequency noise ${S}_{{y}_{\delta {\rm{P}}}}(\omega ,\lambda )$ originates from the intensity fluctuations of the light source employed for photothermal sensing, as well as any other light source used for transduction, such as interferometric lasers^49^. For a continuous wave (CW) source with a power fluctuation PSD *S*_*P*_(*ω*, *λ*) [W^2^/Hz] (see Fig. 4c), the resonator fractional frequency fluctuations are given by

39 $${S}_{{y}_{\delta {\rm{P}}}}(\omega ,\lambda )=\,{\alpha }^{2}(\lambda )\,{{\mathcal{R}}}_{{\rm{P}}}^{2}(\omega )\,{S}_{P}(\omega ,\lambda ),$$

where *S*_*P*_(*ω*, *λ*) typically has the form

40 $${S}_{P}(\omega ,\lambda )={h}_{0}+{h}_{-1}{f}^{-1}+{h}_{-2}{f}^{-2}$$

for a generic laser source^54^. Here, *h*_0_ denotes the laser shot-noise limit ${S}_{P,{\rm{sn}}}(\lambda )=2hc\left\langle {P}_{0}\right\rangle /\lambda$, where $\left\langle {P}_{0}\right\rangle$ is the average input power; the terms *h*_−1_ and *h*_−2_ express the flicker and random walk noise levels, respectively. It is worth noting that *λ* refers to the wavelength of the transduction laser, as well as all the wavelengths of the light source used as a probe for spectroscopy or radiation sensing applications.

Therefore, high optical absorption and responsivity (3) make the resonator more sensitive to laser intensity noise, highlighting a trade-off between responsivity and frequency fluctuations. This noise can be mitigated by selecting materials with low absorption in the targeted spectral range, or by operating the laser at its shot-noise limit ${S}_{P,{\rm{sn}}}(\lambda )$.

## Experimental results and discussion

The experimental results focus on low-stress SiN resonators and are compared with the theoretical model (for details about the fabrication of the resonators and measurement procedures, see Materials and Methods).

### Strings

Figure 3a shows the optical micrograph of a string resonator measured in this study. Figure 3b shows the measured resonance frequency of SiN strings with varying length. The Q factor of these strings, essential for the theoretical calculations of the additive phase noise (31), has also been measured (for the data, see SI Section S3). Figure 3c displays the experimental thermal time constant (dark red circles) compared to theoretical predictions (4), showing excellent agreement.

Figure 3d compares the theoretical model (3) (black solid lines) with the measured responsivity (dark red circles). The uncertainty band is defined by the uncertainty in material parameters *κ* and *E*. All data points fall within the uncertainty band except for *L* = 2 mm. This discrepancy is consistent with the increased radiative losses caused by high probing optical power (*P*_0_ = 24 − 40 *μ*W). Higher incident powers lead to elevated temperatures at the string’s center, increasing the radiative heat flux $\propto ({T}^{4}-{T}_{0}^{4})$. This results in a nonlinear reduction of ${{\mathcal{R}}}_{{\rm{P}}}$, as well as a reduction in photothermal response time *τ*_th_ (see Fig. 3c).

The power responsivity can be enhanced by reducing the resonator’s thickness *h* and width *w*. On one hand, thinner strings will improve the thermal insulation, due to a reduction in cross-sectional area, as well as in emissivity^55^. On the other hand, narrower strings will reduce the hosting area for particle and molecule spectroscopy. Hence, the width choice is critical for photothermal sensing.

Figure 3e displays the Allan deviation (AD) for a string (green solid curve)^50^. All the acquired AD have been compared with the theoretical model, accounting for the transfer functions (31) of the PLL and SSO tracking schemes^33,48^. A good match is observed between measurements and theory (black solid curve) for integration times *τ* < 0.1 s, where the main noise source is additive in-phase (blue solid curve). For *τ* > 0.1 s, the data depart from the thermomechanical asymptote, with the presence of flicker frequency noise for 0.1 s < *τ* < 1 s, and frequency random walk for *τ* > 1 *s*, attributed to photothermal back-action (see below).

Figure 3f presents the resulting NEP values, evaluated at *τ* = *τ*_th_. For each length, three different chips have been analyzed (black circles). The results demonstrate strings’ high photothermal sensitivity ($0.28-2.5\,{\rm{pW}}/\sqrt{{\rm{Hz}}}$). The plot also displays the theoretical NEP (black solid curve), closely aligning with the measurements. For clarity, the measurements’ mean value and the standard error are plotted for each length, falling within the predicted values. The sensitivity is mainly limited by thermomechanical noise for almost all the lengths. The observed deviations are consistent with the photothermal back-action.

The positive correlation between noise level measured for long integration times (*τ* > *τ*_th_) and power responsivity is evidence for photothermal backaction (39). To investigate this further, the laser relative intensity noise *S*_*P*_(*ω*, *λ*) has been characterized for all the optical powers employed in this study and ${S}_{{y}_{\delta {\rm{P}}}}(\omega ,\lambda )$ evaluated. The results are displayed in Fig. 3e with the purple solid curve, showing excellent agreement with the data for *τ* > 0.1 s. The observed flicker and random walk frequency noises are consistent with the intensity spectral distribution *S*_*P*_(*ω*, *λ*), as clearly shown in Fig. 4a, far above the ultimate laser shot noise limit ${S}_{P,{\rm{sn}}}(\lambda )$ (see Materials and methods for details regarding the experimental setup).

Hence, photothermal back-action frequency noise imposes an upper limit on the probing power used for displacement transduction. On the one hand, high laser power improves the displacement sensitivity $\sqrt{{S}_{z}^{min}}$ [${\rm{m}}/\sqrt{{\rm{Hz}}}$], as shown in Fig. 4b*&*c^56^, reducing the detection coefficient ${{\mathcal{K}}}_{d}$. On the other hand, such a signal enhancement saturates at higher optical power due to the induced frequency noise^49^, with any low-frequency intensity noise, such as mode hopping^57^, directly impacting the resonator stability^54^.

Figure 4c further shows that the displacement sensitivity is here inversely proportional to the optical power *P*_0_, indicating that the laser noise has a classical (detector and technical noise) and not quantum shot noise origin^58^. Among the different approaches to mitigate laser classical noise, active intensity stabilization could offer a simple way to push the laser to its shot-noise limit^59^. A simpler approach to reduce the transducing laser-induced photothermal back-action is the use of another wavelength, for which SiN absorption is reduced (e.g. 1550 nm as shown in Ref. ^33^), along with employing a more stable lasing source.

### Drumheads

**Fig. 5:**
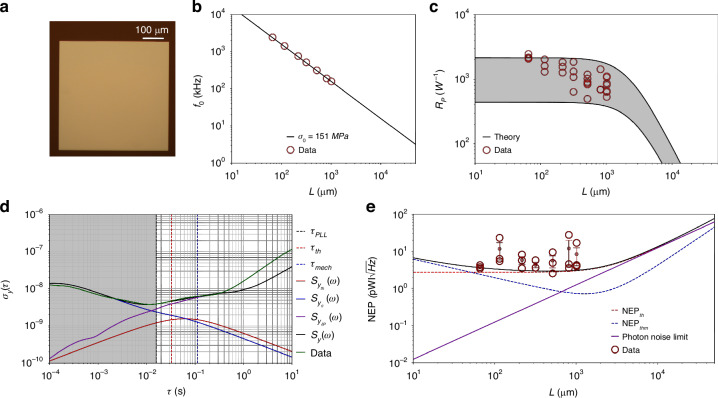
Drumhead design. **a** Optical micrograph of a drumhead resonator. **b** Resonance frequency for 50 nm thick square drumhead resonator of different side length. From the measurements, a stress of 150 MPa is extracted. **c** Comparison between theoretical (solid curves) and measured (dark red unfilled dots) relative power responsivity. The error bar indicates the uncertainties in *κ* (2.7 − 4 W/(m K)), *E* (200 − 300 GPa), and *α*_th_ (1 − 2.2 ppm/K)^16^. **d** Allan deviation measured for a 1 mm^2^ square membrane (green solid curve), driven at the onset of nonlinearity ${z}_{{{\rm{r}}}_{{\rm{c}}}}$, with low-pass filter bandwidth ${f}_{{\rm{demod}}}=2.5$ kHz, PLL bandwidth *f*_PLL_ = 10 Hz and optical input power *P*_0_ = 6 *μ*W. The comparison with the theoretical model is also shown (black solid curve), together with the single contributions (see main text). **e** Comparison between the theoretical (black solid curve) and experimentally extracted (black unfilled dots) NEP for membranes. Temperature (red dashed curve) and thermomechanical (blue dashed curve) fluctuations-induced fractional frequency noise are also shown, together with the single photon noise limited NEP. For each membrane’s length, three different resonators were characterized in terms of NEP

Figure 5a shows the optical micrograph of a drumhead resonator measured in this study. Figure 5b shows the resonance frequency corresponding to the drumheads characterized experimentally (for the measured Q, see SI Section S3). Experimental results concerning the thermal time constant are not presented here, as the photothermal response time of SiN drumheads has been already experimentally characterized elsewhere^30,32^.

Figure 5c compares the theoretical predictions (3) (solid curves) with the experimental data (dark red circles) for the relative power responsivity. The uncertainty band, defined by uncertainties in *κ*, *E*, and *α*_th_, encompasses all the experimental points, indicating a strong agreement between theory and experiments. Figure 5d illustrates the AD for a drumhead. In detail, two regimes can be recognized: i) for different integration times *τ* < 0.01 s, the AD is limited by additive phase noise ${S}_{{y}_{\theta }}(\omega )$ (blue solid curve); ii) *τ* > 0.01 s, the noise is dominated by photothermal backaction ${S}_{{y}_{\delta {\rm{P}}}}(\omega )$. Notably, in the absence of photothermal backaction, temperature fluctuation frequency noise would dominate. This condition, where a mechanical resonator interacts with the environment at the single shot noise level, is of significant interest for micromechanical thermal detectors^5,7,29,33,43^.

Figure 5e presents the experimental NEP evaluated at *τ* = *τ*_th_, alongside the theoretical sensitivity (black solid curve), closely aligning to each other. The experimental results of $1-20\,{\rm{pW}}/\sqrt{{\rm{Hz}}}$ are one order of magnitude lower than previously characterized, electrodynamically transduced drumhead resonators^5^, showing the outstanding performances of pristine SiN structures over integrated nanoelectromechanical systems (NEMS), where electrodes are an important part of the design^5,21,39^. The use of pure SiN for photothermal sensing applications is enabled by noninvasive transduction approaches, such as interferometry. In particular, pure optical transduction offers two key advantages: i) the absence of metal traces increases the thermal insulation, improving the responsivity (3); ii), the sensor is not limited by Johnson noise, which usually degrades the frequency stability (25) of a vast group of NEMS resonators^5^. Conversely, bare SiN drumheads are mainly affected by temperature fluctuations noise (dark red dashed curve), as shown for *L* > 50 *μ*m. Moreover, as the resonator enters the radiation-limited regime, thermal photon shot noise becomes dominant (dark violet solid curve)^32,43^.

### Trampolines

**Fig. 6:**
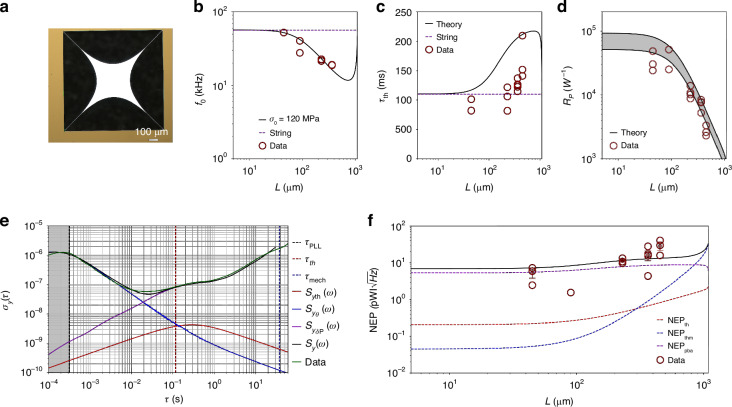
Trampoline design. **a** Optical micrograph of a trampoline resonator. **b** Resonance frequency for 50 nm thick trampoline resonators of different central area side lengths. The window side length is fixed to *L*_w_ ≈ 1 mm, while the tether width is to *w* = 5 *μ*m. From the measurements, a stress of 120 MPa is extracted. **c** Comparison between theoretical (black solid curve) and experimental thermal time constant *τ*_*th*_. **d** Comparison between theoretical (solid curves) and measured (dark red circles) relative power responsivity. The error bar indicates the uncertainties in *κ* (2.7 − 4 W/(m K)), *E* (200 − 300 GPa), and *α*_th_ (1 − 2.2 ppm/K)^16^. **e** Allan deviation measured for a 230^2^ *μ*m^2^ central area trampoline (green solid curve), driven at the onset of nonlinearities ${z}_{{{\rm{r}}}_{{\rm{c}}}}$, with low-pass filter bandwidth ${f}_{{\rm{demod}}}=2.5\,{\rm{kHz}}$, PLL bandwidth *f*_PLL_ = 500 Hz and optical input power *P*_0_ = 11 *μ*W. The comparison with the theoretical model is also shown (black solid curve), together with the single contributions (see main text). **f** Comparison between the theoretical (black solid curve) and experimentally extracted (dark red circles) NEP. For each trampoline’s central length, three different resonators were characterized in terms of NEP

The experimental analysis has been carried out for trampoline resonators with central pads designed using a Bezier profile (see Fig. 6a)^4,5,39–41^, a thickness *h* of 50 nm, a tethers’ width *w* of 5 *μ*m, and tether’s length *L*_t_ ranging from 460 to 756 *μ*m.

Figure 6b presents the resonance frequency as a function of the central area *L*^2^ (for the Q measurements, see SI Section S3). For small areas (*L*^2^ < 50^2^ *μ*m^2^), *ω*_0_ can be approximated with that of a string^42^. In the intermediate range (50^2^ *μ*m^2^ < *L*^2^ < 500^2^ *μ*m^2^) the effective mass *m*_eff_ grows faster (∝ *L*^2^) than the tethers’ effective stiffness *k*_eff_ (∝ *L*^*ζ*^, with *ζ* < 2), leading to a reduction in resonance frequency *ω*_0_. For larger areas (*L*^2^ > 500^2^ *μ*m^2^) *k*_eff_ increases more rapidly than *m*_eff_ (*ζ* > 2), causing *ω*_0_ to rise beyond the string value (see SI Section S4).

Figure 6c compares the theoretical thermal response time (black solid curve) with the experimental measurements (dark red circles). Discrepancies between the model and experimental data may stem from variations in material parameters, as supported by findings in the literature^60^. For instance, differing values of specific heat capacity and mass density from those used in the model would affect the heat capacitance *C*, while variations in emissivity and thermal conductivity would influence the thermal conductance *G*. Nevertheless, a positive correlation between *τ*_th_ and *L* is evident. This slow thermal response significantly impacts the frequency noise in the experimental setup employed here.

Figure 6d shows the comparison between the theoretical and measured power responsivity, exhibiting excellent agreement. As for the other designs, the shaded band represents uncertainties in *κ*, *E*, and *α*_th_.

Figure 6e shows the AD for a trampoline. Also here, two regimes can be recognized: an additive phase noise-limited region for integration times *τ* < 0.02 s, and a fully photothermal back-action frequency noise-dominated region for *τ* > 0.02 s. The sum of all the contributions (black solid curve) is a good match with the experimental data (green solid). It is worth noting that *τ*_th_ lies far in the photothermal back-action dominated region (red dashed vertical line), meaning that, during the time the resonator takes to reach a new thermal equilibrium, e.g. upon energy relaxation by a molecule, intensity fluctuations of the probing laser increase the frequency noise. Conversely, with a shot-noise limited laser, the temperature fluctuation frequency noise would dominate for *τ* > *τ*_th_.

Figure 6f displays the experimental sensitivities evaluated at *τ* = *τ*_th_ (dark red circles) compared with the theoretical calculations (blue and red dashed curves). The plot reveals that the photothermal back-action (dark violet dashed curve) has degraded the sensitivity by one order of magnitude compared to the theoretical expectations. Moreover, this effect is much more pronounced for this design than for the others. Indeed, the slow thermal response time of trampolines makes them more sensitive to the laser relative intensity noise (40), where flicker and random walk noise are present, worsening the corresponding sensitivity

41 $$NE{P}_{{\rm{pba}}}=\alpha \sqrt{{h}_{0}+2\pi {\tau }_{{\rm{th}}}{h}_{-1}+{(2\pi {\tau }_{th})}^{2}{h}_{-2}}.$$

However, the data follow the theoretical trend, with the sensitivity worsening for increasingly larger central areas *L*^2^. As for the *τ*_th_ (Fig. 6c), the observed discrepancies between model and data may arise from variations in material parameters. Similar to drumheads, temperature fluctuations represent the ultimate theoretical limit of the photothermal sensitivity in the absence of photothermal back-action.

### Comparison

**Fig. 7:**
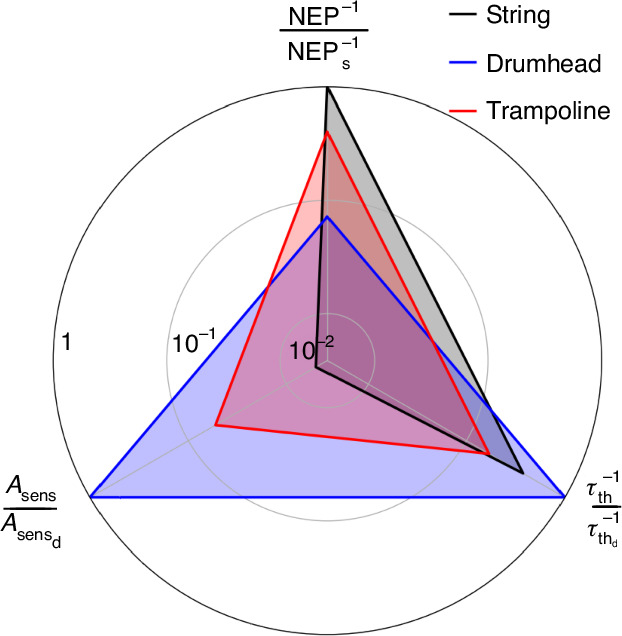
Performance summary. Radar chart of the nanomechanical photothermal performances for a localized heat source. The chart accounts for the normalized NEP, thermal response time *τ*_th_, and sensing area *A*_sens_. The highest value for each metric has been used for normalization, with the subscripts referring to the corresponding design (s, string; d, drumhead; t, trampoline). The string length, the membrane side length, and the trampoline window side length are all 1 mm long. The trampoline has a central area *L*^2^ = 230^2^ *μ*m^2^. All the structures are assumed to be 50 nm thick

In summary, a theoretical comparison among the three resonator designs of comparable dimensions is illustrated in the radar chart shown in Fig. 7. The metrics used for this comparison are the NEP, the thermal time constant *τ*_th_, and the sensing area *A*_sens_, each normalized to the best-performing value.

The string demonstrates the highest photothermal sensitivity due to its superior thermal insulation, albeit with the smallest sensing area. It presents an intermediate thermal response time compared to the other geometries. The fundamental frequency noise limit for this design is likely dominated by thermomechanical phase noise. These features make strings an excellent workhorse for nanomechanical photothermal spectroscopy^28^. Conversely, the drumhead exhibits the lowest sensitivity but offers the largest sensing area and the fastest thermal response. In particular, the combination of high speed and optimal sensitivity for this design makes drumheads ideal for applications requiring quick measurements. Furthermore, temperature fluctuations are expected to be the ultimate frequency noise limit.

Drumheads are good candidates for scanning spectromicroscopy, as well as a promising platform for room-temperature IR/THz detection. In particular, in the case of single-photon noise-limited detection, the large sensing area *A*_sens_ could enable drumheads to achieve the room-temperature specific detectivity limit ${D}^{* }\equiv \sqrt{{A}_{{\rm{sens}}}}/NEP\approx 2\cdot 1{0}^{10}\,{\rm{cm}}\sqrt{{\rm{Hz}}}/W$^5,29,32^. It is important to note that in the regime limited by temperature fluctuations, where ${S}_{y}(\omega )\approx {S}_{{y}_{{\rm{th}}}}(\omega )\propto {A}_{{\rm{sens}}}^{-1/2}$, the NEP increases with the sensing area, following the relationship NEP$\propto \sqrt{{A}_{{\rm{sens}}}}$, reducing the sensitivity of the resonator. Consequently, the specific detectivity D^*^ becomes independent of the area *A*_sens_. In this regime, strings and drumheads present similar performances in terms of specific detectivity (see SI Section S6). However, drumheads offer the advantage of less stringent optical requirements, needing less precise focusing of the incoming IR light and experiencing reduced influence from photothermal back-action.

Trampolines present a compromise between the highly sensitive strings and the drumheads with a larger sensing area. As such, trampolines show intermediate values in terms of power sensitivity and sensing area. Their only drawback is the slow thermal response, which makes them more susceptible to photothermal back-action frequency noise than the other designs, as confirmed by experimental observations. Despite this, their high sensitivity makes this design a good candidate for photothermal spectroscopy. Moreover, temperature fluctuations are expected to be the ultimate limiting frequency noise, therefore making them a promising alternative for IR/THz thermal detection and a potential competitor for drumheads.

The present study has examined the three main resonator designs exploited so far in nanomechanical photothermal sensing. Various optimization methods are available to improve the current state-of-the-art photothermal sensitivity. A straightforward approach is the reduction of the resonator’s thickness *h*, as the $\,\text{NEP}\,={S}_{y}^{1/2}(\omega )/{{\mathcal{R}}}_{{\rm{P}}}\propto \sqrt{h}$. On the one hand, the power responsivity scales with the thickness as ${{\mathcal{R}}}_{{\rm{P}}}\propto {G}^{-1}\propto {h}^{-1}$, since both *G*_cond_ and *G*_rad_ are ∝ *h* (for $h={\mathcal{O}}(10{\rm{nm}})$, the emissivity can be approximated as *ϵ*_rad_ ∝ *h*^55^). On the other hand, the fractional frequency noise scales as *S*_*y*_(*ω*) ∝ *h*^−1^, since both the additive phase noise ${S}_{{y}_{\theta }}(\omega )\propto {\alpha }_{{\rm{Duff}}}{m}_{{\rm{eff}}}^{-2}\propto {h}^{-1}$ and the temperature fluctuations frequency noise ${S}_{{y}_{{\rm{th}}}}(\omega )\propto {G}^{-1}\propto {h}^{-1}$ scale in the same manner.

Beyond thickness optimization, new designs routinely employed in other fields of nanomechanics, e.g., in optomechanics, could be explored for photothermal sensing. For instance, phononic crystal (PnC) engineering could be easily integrated within the sensor, enhancing the resonator’s photothermal response, as well as its thermal properties. In particular, the use of PnC defect flexural modes for sensing applications would boost the power responsivity due to the increased overlap between the photothermally induced temperature field and the mechanical mode volume, as already shown^61^. Exploring resonance modes beyond flexural modes (0.1 − 100 MHz), which usually lie at higher frequencies (> 100 MHz), offers an intriguing direction for further research. High-frequency oscillations could improve the frequency stability of the resonator, especially against thermomechanical noise. This approach would likely involve materials other than SiN, such as lithium niobate, which additionally supports design integration capabilities^62^.

## Conclusions

In summary, the comparative analysis conducted on three distinct resonator designs utilized in photothermal sensing-namely strings, drumheads, and trampolines-has elucidated the relationship between the resonator’s photothermal sensitivity and its mechanical and thermal properties. Across all scenarios, the theoretical framework has shown remarkable consistency with both experimental data and FEM simulations, demonstrating how the resonance frequency photothermal response is governed by the resultant mean temperature rise. Overall, strings emerge as the most sensitive design, followed by trampolines and drumheads. Conversely, drumheads exhibit the fastest thermal response, followed by strings and trampolines. The analysis has also highlighted the critical role of photothermal back-action, particularly its impact on the trampolines’ frequency fluctuations, due to their slowest thermal response. Therefore, high photothermal sensitivity can be achieved with low-tensile-stressed, thin resonators, especially when combined with low-noise detection methods like interferometry. For optical readouts particularly, utilizing low-intensity lasers and low-absorption materials will be crucial in minimizing photothermal back-action.

The findings reported here not only clarify the relative performance of the resonator designs investigated but also establish a solid groundwork for the development of next-generation nanomechanical photothermal sensors. This study contributes to the advancement of nanomechanical sensing technology, offering valuable insights for researchers seeking to harness the full potential of photothermal sensing in diverse applications.

## Materials and methods

### Fabrication of nanomechanical resonators

The tensile stressed silicon nitride (SiN) resonator were fabricated with a low-pressure chemical vapor deposition (LPCVD) process on a double-sided ≈ 50 nm thick SiN on $\left\langle 100\right\rangle$ silicon (Si) wafer. Photolithography was used to pattern both the front and back sides of each chip according to the specific design. The structures were then released through a KOH etching process, which removed the underlying Si substrate^16,61^. For each design, the in-plane dimensions where characterized by optical microscopy, and the thickness was measured with ellipsometry.

### FEM simulations

FEM analysis (COMSOL Multiphysics, v5.5 and v6.1) has been carried out to determine the relative power responsivity ${{\mathcal{R}}}_{{\rm{P}}}(0)$ and the thermal time constant *τ*_th_ of the resonators. For ${{\mathcal{R}}}_{{\rm{P}}}(0)$, the Solid Mechanics Physics has been employed in conjunction with Heat Transfer in Solids. First, a Static Prestress Study is performed to solve for the resonator stress field, accounting for the initial prestress *σ*_0_ and the thermal stress components induced by laser heating. A Gaussian beam with beam waist *w*_0_ is used as the light source. The static solutions obtained from this study serve as input parameters for the Eigenfrequency Study, where the fundamental eigenfrequency is computed. The procedure is repeated for different input powers *P*_0_. For the evaluation of *τ*_th_, a time-dependent study is conducted with the beam impinging at the resonator center. The temperature of the resonator is solved for discrete points in time, and the thermal time constant is evaluated upon fitting with an exponential function. More details are given in Section S1 in the Supporting Information.

### Experimental setup

The resonators are operated in high-vacuum conditions (*p* < 10^−5^ mbar) to minimize gas damping and thermal convection, in a custom-designed vacuum chamber equipped with a window for optical access to the chips. The resonators are actuated with a piezoelectric element placed beneath them. Their out-of-plane displacement is measured with a commercial laser-Doppler vibrometer (MSA 500, Polytec GmbH), operated at 633 nm wavelength with a HeNe laser. The vibrometer’s signal is sent to a lock-in amplifier (HF2LI, Zurich Instruments) equipped with a PLL module, or to a frequency counter implemented in a self-sustained oscillator (PHILL, Invisible-Light Labs GmbH)^48^. The relative power responsivity ${{\mathcal{R}}}_{{\rm{P}}}(0)$ is evaluated upon measurement of the thermomechanical noise spectrum peak for different input laser powers, as described in^61,63^. The thermal time constant *τ*_th_ is evaluated with the 90/10 method, as described in refs. ^2,5^. For that, the resonator is driven at its resonance frequency with the PLL or SSO tracking scheme. More details are given in Section S4 in the Supporting Information.

### Laser intensity fluctuations characterization

The intensity of the probing laser has been acquired for 1 minute with a silicon photodiode (Thorlabs GmbH S120C, 1 *μ*s response time) together with a digital power meter console (Thorlabs GmbH PM100D). The electrical signal is fed to the lock-in amplifier^49^, with a filter bandwidth of *f*_L_ = 3 kHz. The recorded intensity signal is then converted into frequency fluctuations, accounting for the resonator’s thermal response *H*_th_(*ω*), and the corresponding AD is evaluated (See SI Section S5).

## Supplementary information

Supplementary information
