# Supplementary material for: Comparative analysis of nanomechanical resonators: sensitivity, response time, and practical considerations in photothermal sensing
Source: Microsyst Nanoeng. 2025 Feb 18;11:28. doi: 10.1038/s41378-025-00879-6 (PMC11836225; doi:10.1038/s41378-025-00879-6)
Supplement: Supplementary file 1 — Supplementary information [file 41378_2025_879_MOESM1_ESM.pdf]

Supplementary information: Comparative Analysis of Nanomechanical Resonators: Sensitivity, Response Time, and Practical Considerations in Photothermal Sensing

Kostas Kanellopulos,[1] Friedrich Ladinig,[1] Stefan Emminger,[1] Paolo Martini,[1] Robert G. West,[1] and Silvan Schmid[1,*]

[1] *Institute of Sensor and Actuator Systems, TU Wien, Gusshausstrasse 27-29, 1040 Vienna, Austria.*

(Dated: November 10, 2024)

CONTENTS

S1. FEM simulation workflow 2
A. Power Responsivity $\mathscr{R}_\mathrm{P}$ 2
B. Thermal Time Constant τ_th 3

S2. Theory workflow 4

S3. Mean temperature framework (MTF) 5
A. String 6
1. Shape and β factors 6
2. Heat localization 6
B. Drumhead 7
1. Shape and β factors 7
2. Heat localization 9
C. Trampoline 10
1. Heat localization 10

S4. Mechanics of the trampoline 13

S5. Measurement procedure 15
A. Relative power responsivity $\mathscr{R}_\mathrm{P}$ 15
B. Thermal time constant τ_th 15
C. Frequency noise 15
1. PLL settings 15
2. Experimental Q factors 17
3. Photothermal back-action 18

S6. Specific Detectivity 19

References 20

---

* Correspondence email address: silvan.schmid@tuwien.ac.at

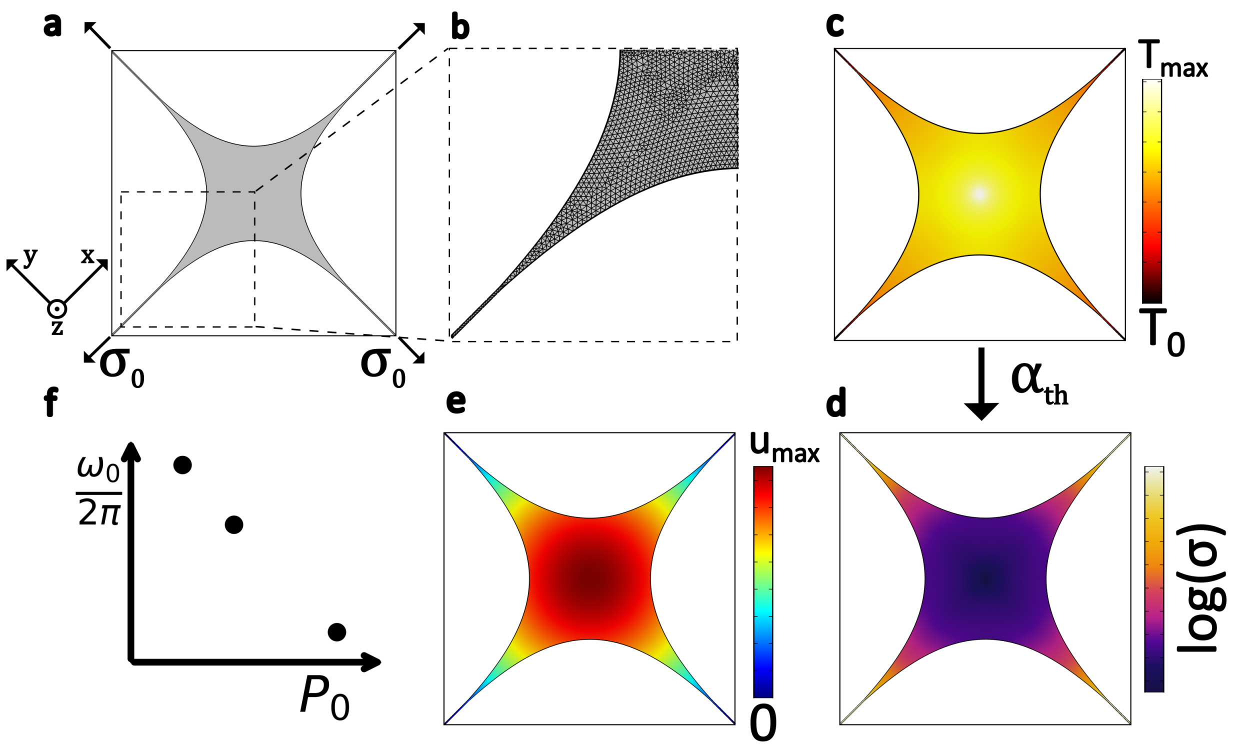

Figure S1. **Photothermal response FEM workflow. a.** Definition of geometry, material properties, and boundary conditions. **b.** Mesh configuration. **c.** Stationary study I: Steady-state temperature field T computed for a given input heat power P_0. **d.** Stationary study II: Resultant (steady-state) static stress field σ, derived from the temperature field in **c**, which couples to σ via the thermal expansion (with the coupling strength determined by the thermal expansion coefficient α_{th}). **e.** Eigenfrequency study: Modeshape of the fundamental out-of-plane flexural mode. **f.** Parametric study: The eigenfrequency is solved for varying input power P_0.

S1. FEM SIMULATION WORKFLOW

The FEM simulations were conducted to study the relative power responsivity $\mathscr{R}_{\text{P}}$ and thermal time constant τ_{th} using Structural Mechanics Module and Heat Transfer Module available in COMSOL have been used. The Structural Mechanics Module handles statics, eigenfrequency, and transient analyses, while the Heat Transfer Module addresses heat conduction, convection, and radiation in transient or steady-state regimes. The modules are coupled through thermal expansion in the resonator caused by heat interaction. Material properties are assumed constant across simulated temperatures.
The simulation setup starts with defining the resonator's geometry and materials (Fig. S1a). Designs have high aspect ratios ($L/h > 10^3$, with $h = 50$ nm), enabling a 2D shell interface that reduces the problem from 3D to 2D, with thickness h included as input parameter in motion and heat transfer equations.
For mechanical analysis, the material is modeled as linear and elastic with Young's modulus E, Poisson's ratio ν, and density ρ. A uniform tensile stress $\sigma_{xx} = \sigma_{yy} = \sigma_0$ is applied, and clamping regions are fixed to ensure zero displacement at the edges. Thermal parameters include the thermal expansion coefficient α_{th}, specific heat c_p, thermal conductivity κ, and emissivity ε_{rad}. The heat source can be a Gaussian laser beam or boundary source with power P_0, with no convection, as experiments are in vacuum. For the heat transfer problem, temperature at the clamping regions is fixed at $T_0 = 300$ K to model thermal bath coupling. Meshing follows (Fig. S1b), using quadrilateral or triangular elements depending on design. Mesh refinement balances computational efficiency with accurate physics representation.

A. Power Responsivity $\mathscr{R}_{\text{P}}$

First, the steady-state temperature profile $T(x,y)$ within the resonator is computed (Fig. S1c). Next, using $T(x,y)$ as input, the static stress field $\sigma(x,y)$ is determined (Fig. S1d). Thermal expansion, of coefficient α_{th}, links temperature and stress through thermal relaxation. Finally, the fundamental eigenfrequency (Fig. S1e) is computed, using the stress field as input. These steps

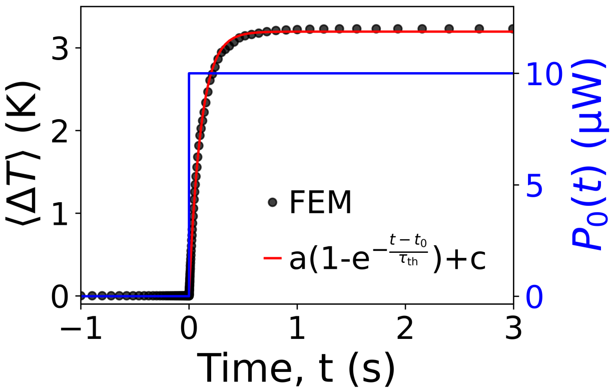

Figure S2. **FEM thermal time constant.** The FEM results (black dots) are fitted with an exponential function of the form $a(1-e^{-\frac{t-t_0}{\tau_{\text{th}}}})+c$.

are repeated for different laser powers P_0, and responsivity $\mathscr{R}_P$ is extracted by fitting the FEM results (Fig. S1f) with

$$\frac{\omega_0(P_0)}{2\pi} = [1+\alpha(\lambda)\,\mathscr{R}_\text{P}(\omega)\,P_0]\,\frac{\omega_0(0)}{2\pi}, \tag{S1}$$

with $\omega_0(0)/2\pi$ denoting the resonator's eigenfrequency for no impiging optical power ($P_0 = 0\ \mu$W).
Within these simulations, it is also possible to access the heat flux due to conduction and radiation at step 3 (Fig. S1c) from the Heat Transfer Module, from which the thermal conductance is computed and compared with the theoretical model.

B. Thermal Time Constant τ_{th}

Two simulation types were implemented to evaluate τ_{th} through a transient thermal study where the laser is turned on at time $t = 0$ to solve the temperature profile evolution over time (Fig. S2). The first study tracks the maximum and surface-averaged temperatures of the resonator, assessing how quickly it reaches steady-state. The second study extends this by using the temperature profile as input for mechanical simulations, similar to $\mathscr{R}_P$. For each time step $t > 0$, the temperature field $T(x,y)$ and corresponding tensile stress field are updated to evaluate the eigenfrequency evolution. Both simulations are fitted with an exponential function, $a(1-e^{-\frac{t-t_0}{\tau_{\text{th}}}})+c$, as shown in Fig. S2.

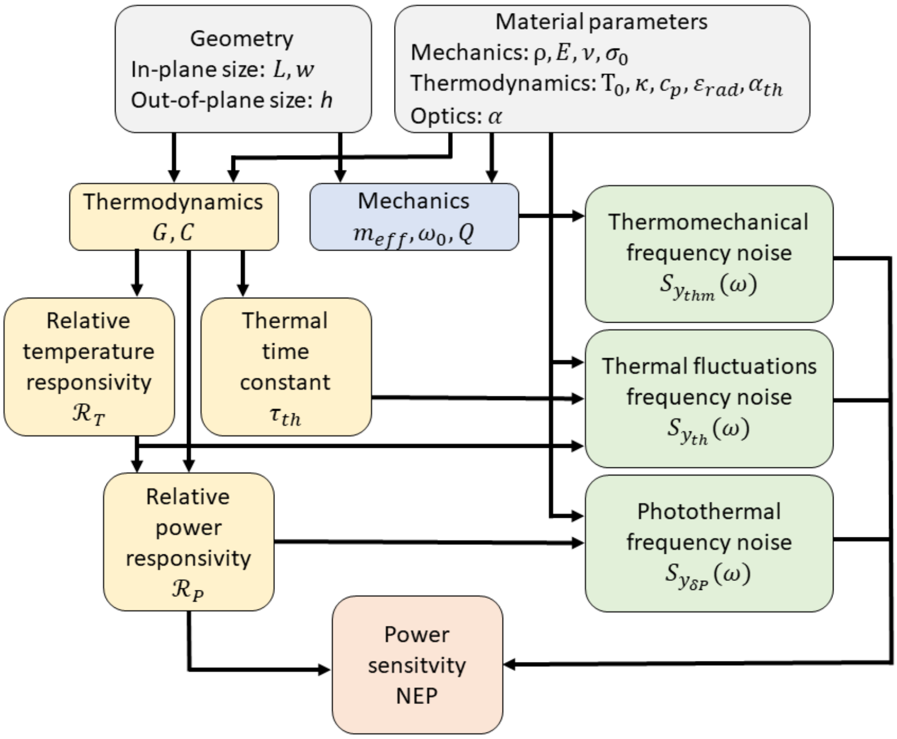

Figure S3. **Theory workflow.** Schematics of the workflow for the theoretical calculations of the power responsivity $\mathscr{R}_P$, frequency noise $S_y(\omega)$, and the power sensitivity NEP.

S2. THEORY WORKFLOW

The theoretical calculation workflow for the power responsivity $\mathscr{R}_P$, frequency noise $S_y(\omega)$, and the power sensitivity NEP is depicted in Fig. S3. Intially, he geometric and material parameters are defined (the values used in this work has provided in the figure captions). Next, the effective mass m_{eff}, resonance frequency ω_0 and the Q factor are established for the mechanical properties. Simultaneously, the thermal conductance (Eq. (15)) and capacitance (Eq. (14)) are determined for the thermodynamic properties. These thermal parameters are then used to calculate the relative temperature responsivity $\mathscr{R}_T$ (Eq. (8),(10) or (12)) and the thermal time constant τ_{th} (Eq. (4)), leading to the computation of the relative power responsivity $\mathscr{R}_P$ (Eq. (3)).
Regarding frequency noise, the thermochanical noise $S_{y_{\text{th}}}$ (Eq. (31)) is derived from the mechanical properties, while the thermal fluctuations noise (Eq. (35)) is calculated based on the thermal characteristics. The photothermal back-action noise (Eq. (39)) is obtained using the power responsivity and the material's optical absorption.
Finally, the power sensivitiy NEP (Eq. (2)) is evaluated.

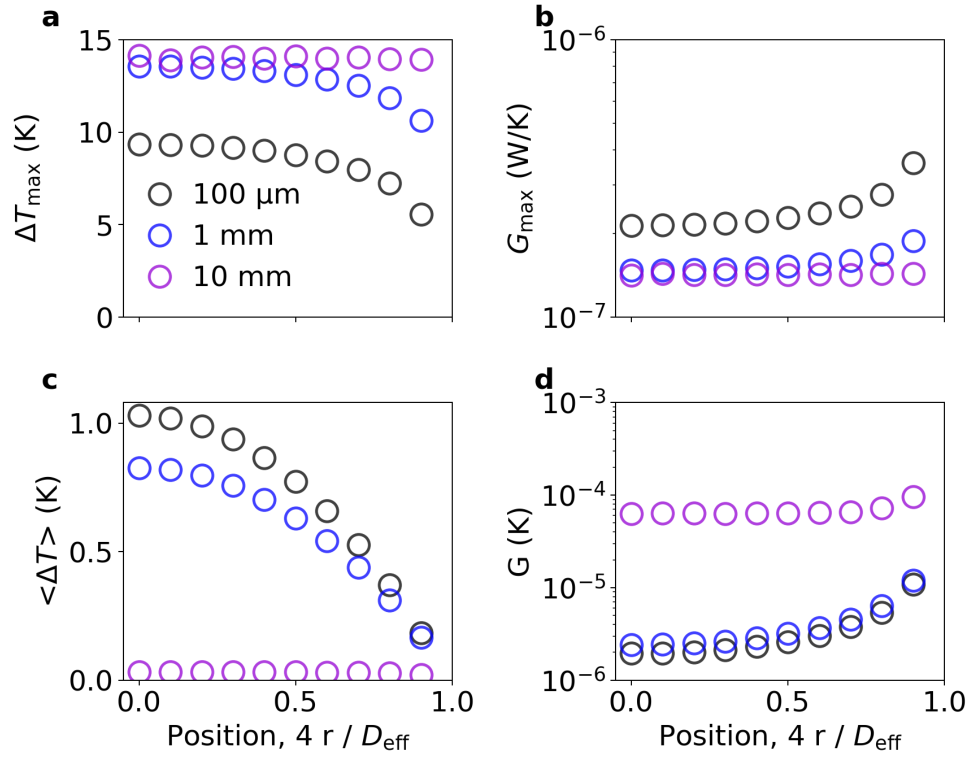

Figure S4. **Mean temperature framework.** FEM simulations of drumhead resonators heated by a laser of power $P_0 = 10$ μm and beam waist of 1 μm, for different resonator size: 100 μm, black empty dots; 1 mm, blue; 10 mm, dark violet. **a.** Maximum temperature rise $\Delta T_{\rm max}$ as a function of the laser position. **b.** Corresponding thermal conductance $G_{\rm max} = P_0/\Delta T_{\rm max}$. **c.** Mean temperature $\langle \Delta T \rangle$ for the same laser conditions. **d.** Corresponding thermal conductance $G = P_0/\langle \Delta T \rangle$.

S3. MEAN TEMPERATURE FRAMEWORK (MTF)

The resonance frequency is a global property of a resonator, depending on its material and geometry. Consequently, variations in resonance frequency are expected to be dictated by the mean temperature changes $\langle \Delta T \rangle$, rather than the local variations ΔT. This has been clearly shown in Fig. 2h. To further underline this point, FEM simulations for circular drumheads of different sizes ($L = 100$ μm, black; 1 mm, blue; 10 mm, dark violet) are performed with a tightly focused heating laser, for different laser positions (Fig. S4). Fig. S4a&b display the FEM results for the maximum temperature rise $\Delta T_{\rm max}$ at thermal equilibrium, and the corresponding thermal conductance $G_{\rm max} = P_0/\Delta T_{\rm max}$, respectively. For the concentric case ($r = 0$), $\Delta T_{\rm max}$ increases for larger drumheads, making the largest resonator the most thermal insulated. Fig. S4c&d display the mean temperature rise $\langle \Delta T \rangle$ and corresponding thermal conductance $G = P_0/\langle \Delta T \rangle$ for the same FEM simulations, respectively. Again, looking only at the concentric case, these results show a completely opposite trend than what it is shown in Fig. S4a&b: the mean temperature rise at thermal equilibrium reduces with larger resonators, leading to an increase in conductance with the drumhead's lateral dimensions. Furthermore, the simulated orders of magnitudes of G are consistent with the power responsivity analysis discussed in the main text (see Fig. 2g). Hence, the mean temperature framework (MTF) must be employed to accurately describe the photothermal response of the resonator. Within this framework, the shape s_f and the β factors must be introduced. These quantities redefine the thermal losses due to heat conduction in the MTF as

$$G_{\rm cond} = \frac{s_f(\mathbf{r}, L, w_0)}{\beta(\mathbf{r}, L, w_0)}\kappa, \tag{S2}$$

with $\mathbf{r} = (x, y)$ or (r, θ), L, and w_0 denoting the heat source position vector, the resonator characteristic length, and the heat source size, respectively.

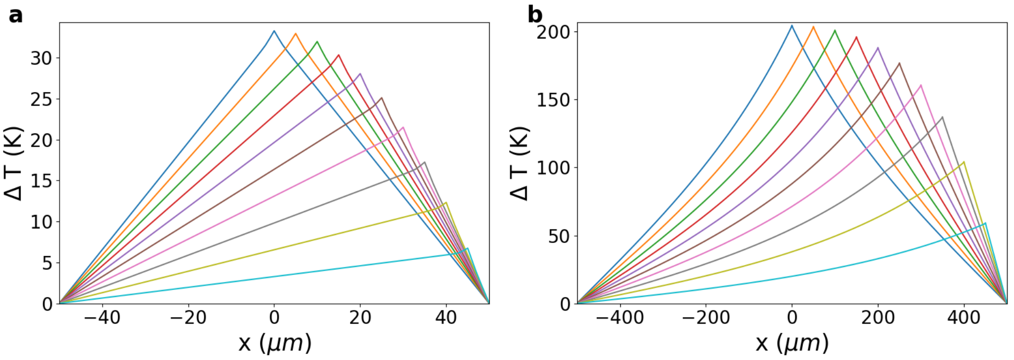

Figure S5. **String temperature profile.** **a** FEM simulated 1D temperature profiles of a 100 μm long string resonator, for different positions of the heat source. **b** FEM simulated 1D temperature profiles for a 1 mm long string. Laser parameters: input optical power $P_0 = 200$ μW, beam waist $w_0 = 1$ μm. FEM parameters: $\rho = 3000$ kg/m^3, $c_p = 700$ J/(kg K), $\kappa = 3$ W/(m$\cdot$K), $E = 250$ GPa, $\sigma_0 = 200$ MPa, $\nu = 0.23$, $\alpha_{\mathrm{th}} = 2.2$ ppm/K, $\varepsilon_{\mathrm{rad}} = 0.05$, $\alpha_{\mathrm{abs}} = 0.5$ %, $w = 5$ μm, $h = 50$ *nm*.

A. String

1. Shape and β factors

The string design represents the simplest geometry from a thermal transport standpoint. Considering a tightly focused laser as heat source, impinging with power P_0 in a position x along the length of the resonator in thermal equilibrium with it, the Fourier law's gives [1]

$$P_0 = \frac{4wh}{L}\kappa\Delta T_{\max} = s_f(x, L, w_0)\kappa\Delta T_{\max}, \tag{S3}$$

with w, h, and L being the resonator width, thickness and length, respectively. $\Delta T_{\max} = T_{\max} - T_0$ denotes the peak temperature rise with respect to the frame temperature T_0, occurring at the heat source position. For a string resonator, such as those analyzed in the main text, a linear temperature profile is the solution of the heat diffusion equation in steady-state for short and intermediate length ($L \leq 2$ mm)

$$\Delta T(x) = \Delta T_{\max} - \frac{2}{L}\Delta T_{\max}|x|, \quad \text{for } -\frac{L}{2} \leq x \leq \frac{L}{2}, \tag{S4}$$

as shown in Fig. S5. The 1D temperature profiles for 100 μm (left) and 1 mm (right) long strings have been obtained using FEM for different heating laser positions. For both strings, all profiles are linear. In the 1 mm long string, the thermal radiation plays a more significant role than in the 100 μm long one, causing the profile to deviate slightly from a purely linear trend. Nonetheless, as long as ΔT can be treated as a linear function of the position $\mathbf{r}$, even in the presence of thermal radiation losses, the following geometrical relation holds true

$$\langle \Delta T \rangle = \frac{1}{L}\int_{-L/2}^{L/2} \Delta T(x)dx = \frac{1}{L}\frac{L\Delta T_{\max}}{2} = \frac{\Delta T_{\max}}{2}, \tag{S5}$$

yielding the β factor for a string resonator as $\beta = \frac{1}{2}$.

2. Heat localization

Nanomechanical photothermal sensing can be performed with tightly focused as well as uniformly distributed heat sources / beam diameters. Greater (lesser) localization of the heating yields higher (lower) temperature rises $\langle \Delta T \rangle$. For a string resonator, two types of FEM simulations have been carried out: i) local heating (LH) with a point heat source at the string's center; ii) uniform heating (UH) with the upper surface defined as the heating source. No Gaussian beam lasers are used here, since part of the total input power would be lost in the uniform heating condition, perpendicular to the string length. The ratio $\mathscr{R}_P^{LH}/\mathscr{R}_P^{UH}$ between the LH and UH power responsivity is plotted as a function of the string length in Fig. S6. For $L \leq 1$ mm, this ratio is constant at 1.5, indicating that localized heating provides a 1.5$\times$ improvement in power responsivity compared to uniform

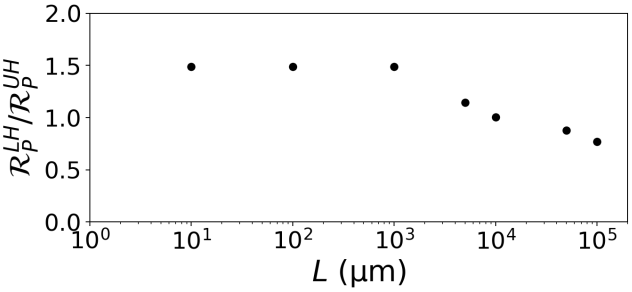

Figure S6. **Heat localization in strings.** Ratio between power responsivity for localized (LH) and uniform (UH) heating conditions. FEM parameters: $\rho = 3000$ kg/m^3, $c_p = 700$ J/(kg K), $\kappa = 3$ W/(m$\cdot$K), $E = 250$ GPa, $\sigma_0 = 200$ MPa, $\nu = 0.23$, $\alpha_{\text{th}} = 2.2$ ppm/K, $\varepsilon_{\text{rad}} = 0.05$, $\alpha_{\text{abs}} = 0.5$ %, $w = 5$ μm, $h = 50$ nm, $P_0 = 10$ μW.

heating. For longer strings ($L > 1$ mm), the highly localized optical power at the center increases the radiation losses $\propto (T^4 - T_0^4)$, worsening the responsivity improvement.

For uniform illumination, all the points along the string length will contribute to the heat dissipation. Integrating (S2) for a string, for a concentric source gives

$$G_{\text{cond}} = \left(\frac{1}{\kappa} \frac{1}{L} \int_0^L \frac{\beta}{s_{\text{f}}(x)} \text{d}x \right)^{-1} = 12 \frac{wh}{L} \kappa. \tag{S6}$$

The overall conductance for a uniformly heated string is given by

$$G = 12 \frac{wh}{L} \kappa + 8 w L \varepsilon_{\text{rad}} \sigma_{\text{SB}} T_0^3 \tag{S7}$$

B. Drumhead

1. *Shape and β factors*

For simplicity, a circular membrane of diameter $D_{\text{eff}} = 2L/\sqrt{\pi}$ [2] is considered here, heated by a laser source of beam waist w_0 centered at position (r, θ) relative to the membrane center (Fig. 2e). Given that $h << D_{\text{eff}}$, no thermal gradient along the resonator thickness is present, consistent with the eccentric shell scenario [3]. For the specific case where the temperature is constant within the source region (red dashed curve in Fig. S7a), the temperature profile is given by

$$\Delta T(r) = \begin{cases} \frac{P_0}{4\pi\kappa h} \ln\left(\frac{D_{\text{eff}}^2}{4w_0^2}\right) & \text{for } 0 \leq r < w_0 \\ \frac{P_0}{4\pi\kappa h} \ln\left(\frac{D_{\text{eff}}^2}{4r^2}\right) & \text{for } w_0 \leq r \leq \frac{D_{\text{eff}}}{2}. \end{cases} \tag{S8}$$

For this scenario, an analytical solution is available for the shape factor [3]

$$s_f(r, \theta, D_{\text{eff}}, w_0) = \frac{2\pi h}{\cosh^{-1}\left(\frac{D_{\text{eff}}^2 + 4w_0^2 - 4r^2}{4 D_{\text{eff}} w_0}\right)}. \tag{S9}$$

The resulting dissipated heat is given by $q = s_f \kappa \Delta T(w_0)$. For a laser beam impinging on the drumhead with input power P_0, the resulting temperature profile is given by (light blue solid curve in Fig S7a)

$$\Delta T(r) = \begin{cases} \frac{P_0}{4\pi\kappa h} \left[\left(1 - \frac{r^2}{w_0^2}\right) + \ln\left(\frac{D_{\text{eff}}^2}{4w_0^2}\right)\right] & \text{for } 0 \leq r < w_0 \\ \frac{P_0}{4\pi\kappa h} \ln\left(\frac{D_{\text{eff}}^2}{4r^2}\right) & \text{for } w_0 \leq r \leq \frac{D_{\text{eff}}}{2}. \end{cases} \tag{S10}$$

Eq. (S10) differs from (S8) within the heated region, due to the different boundary conditions. In this case, the corresponding shape factor s_f is obtained by rewriting $\Delta T(w_0)$ as a function of the maximum temperature rise ΔT_{max}. For the simple case of

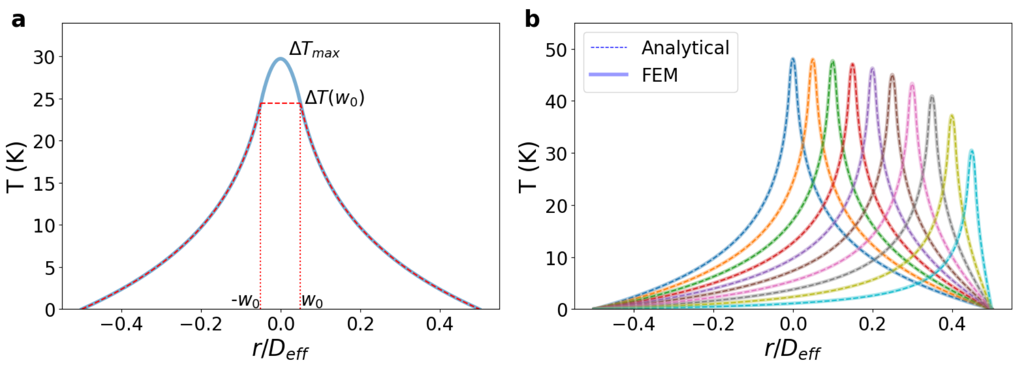

Figure S7. **Membranes temperature profile.** **a** 1D Temperature profile of circular membrane of diameter D_{eff}, heated in the center by a top-hat disk beam of diameter d (light blue solid curve). For comparison, the temperature distribution in the case of a eccentric cylinder, uniformly heated is shown (red dashed curve). **b** Comparison between FEM (solid curves) and analytical (dashed curves) temperature profiles, obtained for a localized heat source of input power $P_0 = 10\ \mu$W and beam waist $w_0 = 1\ \mu$m, moving along a radial cut-line. FEM parameters: $\rho = 3000$ kg/m^3, $c_p = 700$ J/(kg K), $\kappa = 3$ W/(m·K), $E = 250$ GPa, $\sigma_0 = 200$ MPa, $\nu = 0.23$, $\alpha_{\text{th}} = 2.2$ ppm/K, $\varepsilon_{\text{rad}} = 0.05$, $\alpha_{\text{abs}} = 0.5$ %, $h = 50\ nm$.

concentric, conduction limited heat transport problem, this relation is given by [4]

$$\Delta T(w_0) = \frac{P_0}{4\pi\kappa h}\ln\left(\frac{D_{\text{eff}}^2}{4w_0^2}\right) = \Delta T_{\max} - \frac{P_0}{4\pi\kappa h}. \tag{S11}$$

Substituting Eq. (S11) into Fourier's law gives

$$P_0 = s_f(r,\theta,D_{\text{eff}},w_0)\kappa\Delta T(w_0) = \frac{2\pi h}{\cosh^{-1}\left(\frac{D_{\text{eff}}^2+4w_0^2-4\mathbf{r}^2}{4D_{\text{eff}}w_0}\right)}\kappa\left(\Delta T_{\max} - \frac{P_0}{4\pi\kappa h}\right). \tag{S12}$$

Rearranging Eq. (S12) as a function of the peak temperature rise $\Delta T_{\max}$ gives

$$P_0 = \frac{4\pi h}{2\cosh^{-1}\left(\frac{D_{\text{eff}}^2+4w_0^2-4\mathbf{r}^2}{4D_{\text{eff}}w_0}\right)+1}\ \kappa\,\Delta T_{\max} = s_f(r,\theta,D_{\text{eff}},w_0)\kappa\Delta T_{\max}. \tag{S13}$$

Eq. (S13) describes the heat conduction losses with respect to the maximum temperature rise. The analytical solution (S13) has been tested for different heat source positions against FEM simulations, showing excellent agreement. Fig. S7b shows the resulting FEM (solid curves) and analytical (dashed curves) temperature profiles, for an impinging laser power of 10 μW and a beam waist of 1 μm. For the implementation of the MTF, the ratio between mean and peak temperature β must be found. Combining the two expressions of Eq. (S10), it is possible to extract the peak temperature

$$\Delta T_{\max} = \Delta T(0) = \frac{P_0}{4\pi\kappa h}\left[1+\ln\left(\frac{D_{\text{eff}}^2}{4w_0^2}\right)\right] \tag{S14}$$

Integrating Eq. (S10) over the whole resonator area gives the mean temperature

$$\begin{aligned}\langle\Delta T\rangle &= \frac{1}{A}\iint_A \Delta T(r,\theta)\mathrm{d}A = \frac{4}{\pi D_{\text{eff}}^2}\left[\int_0^{2\pi}\int_0^{w0}\Delta T(r)r\mathrm{d}r\mathrm{d}\theta + \int_0^{2\pi}\int_{w_0}^{\frac{D_{\text{eff}}}{2}}\Delta T(r)r\mathrm{d}r\mathrm{d}\theta\right]\\ &= \frac{4}{\pi D_{\text{eff}}^2}\frac{P_0}{4\pi\kappa h}\left[\int_0^{2\pi}\int_0^{w0}\left[-\frac{r^2}{w_0^2}+1+\ln\left(\frac{D_{\text{eff}}^2}{4w_0^2}\right)\right]r\mathrm{d}r\mathrm{d}\theta - \int_0^{2\pi}\int_{w_0}^{\frac{D_{\text{eff}}}{2}}\ln\left(\frac{4r^2}{D_{\text{eff}}^2}\right)r\mathrm{d}r\mathrm{d}\theta\right]\\ &= \frac{4}{\pi D_{\text{eff}}^2}\frac{P_0}{4\pi\kappa h}\pi\left[\frac{w_0^2}{2}+w_0^2\ln\left(\frac{D_{\text{eff}}^2}{4w_0^2}\right)+\frac{D_{\text{eff}}^2}{4}-w_0^2-w_0^2\ln\left(\frac{D_{\text{eff}}^2}{4w_0^2}\right)\right] = \frac{P_0}{4\pi\kappa h}\left(1-\frac{1}{2}\frac{4w_0^2}{D_{\text{eff}}^2}\right).\end{aligned} \tag{S15}$$

Hence, the β factor for the drumhead design can be written as

$$\beta(r,\theta,D_{\text{eff}},w_0) = \frac{1-\frac{1}{2}\frac{4w_0^2}{D_{\text{eff}}^2}}{1+\ln\left(\frac{D_{\text{eff}}^2}{4w_0^2}\right)}\left(1-\frac{4r^2}{D_{\text{eff}}^2}\right), \tag{S16}$$

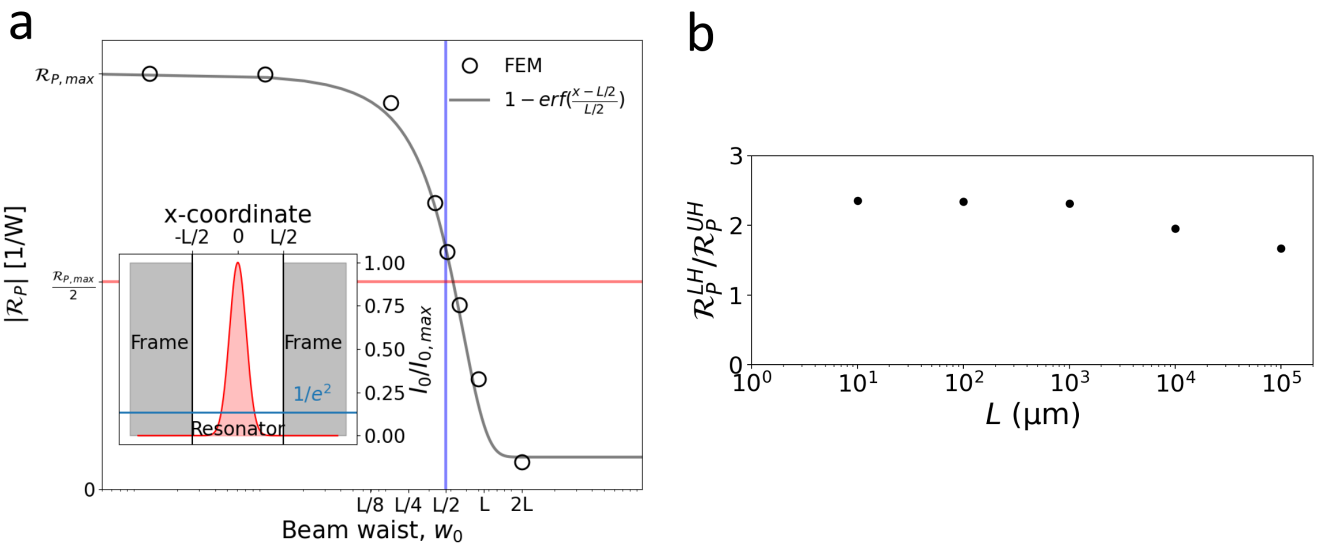

Figure S8. **Heat localization in membranes. a** FEM-aided relative power responsivity of a membrane resonator of side length L, as a function of the light source beam waist w_0. A laser with a gaussian profile is defined as heating source, with a constant input power P_0 and concentric to the resonator. Inset: gaussian beam profile of the input light source. The $1/e^2$ definition has been used for the beam waist w_0. **b** FEM simulated ratio between LH and UH power responsivity as a function of the drumhead side length L. FEM parameters: $\rho = 3000$ kg/m^3, $c_p = 700$ J/(kg K), $\kappa = 3$ W/(m $\cdot$ K), $E = 250$ GPa, $\sigma_0 = 200$ MPa, $\nu = 0.23$, $\alpha_{\mathrm{th}} = 2.2$ ppm/K, $\varepsilon_{\mathrm{rad}} = 0.05$, $\alpha_{\mathrm{abs}} = 0.5$ %, $h = 50$ *nm*.

with the first term denoting the ratio between mean and maximum temperature rise, while the second term expressing the spatial dependence of the β factor. The latter follows by an heuristic approach, by fitting the FEM results.

2. *Heat localization*

FEM simulations have also been conducted for the drumhead resonator to show the dependence of the power responsivity on the localization of the heating source. Fig. S8a shows the FEM analysis for a drumhead of side length $L = 1$ mm. As the beam waist w_0 of a concentric Gaussian beam is increased, $\mathscr{R}_{\mathrm{P}}$ reduces following the complementary error function $1 - erf[(w_0 - L/2)/L/2]$, being the absorbed power the convolution between resonator and laser spot size. For tightly focused beams, all the power is concentrated onto the resonator, resulting in a higher mean temperature increase. For a beam waist $w_0 \simeq L/2$, $\mathscr{R}_{\mathrm{P}} \simeq \mathscr{R}_{\mathrm{P,max}}/2$. This analysis has been carried out also for different drumhead side lengths L, as done for the strings. Fig. S8b shows the FEM results. For $L \leq 1$ mm, the power responsivity for a localized heating source is $\approx 2\times$ higher than for the uniform heating condition. Again, this means in a two-fold improvement in power responsivity. For larger drumheads, the ratio $\mathscr{R}_P^{LH}/\mathscr{R}_P^{UH}$ reduces for the same reason as in strings. Substituting Eq. (S13) and (S16) into equation (S2) for a uniform ($w_0 = D/2$) heating concentric ($r = 0$) to the drumhead gives

$$G_{\mathrm{cond}} = \frac{4\pi h\kappa}{1 + 2\cosh^{-1}(1)} \frac{1 + \ln(1)}{1 - \frac{1}{2}} = 8\pi h\kappa, \tag{S17}$$

meaning that the conductive contribution is doubled. This is clearly show in Fig. S9b. It is also shown here that G_{cond} increases linearly with L for increasingly larger drumheads for uniform heating (dashed blue curve). Indeed, uniformly heated large drumheads will dissipate more heat through the frame. It is important to note that the MTF (black and red dashed horizontal lines) does not accurately capture thermal conduction for drumheads larger than 1 mm, where thermal radiation becomes the dominant mechanism. However, this contribution to the overall thermal conductance G is minimal, enabling an accurate modeling of the complete thermal response even for large drumheads ($L > 1$ mm). Fig. S9c shows the corresponding heat radiation. Both uniformly and locally heated drumheads follow the same trend. Conversely, the relative temperature responsivity changes as a function of the heat localization in drumheads. Indeed, the thermal stress is in general function of the spatial temperature distribution. For a circular drumhead, the position-dependent thermal stress can be written as [2, 4]

$$\sigma(T) = \sigma_0 + \sigma_{\mathrm{th}}(T) = \sigma_0 \left[1 + \frac{\sigma_{\mathrm{th}}(T)}{\sigma_0}\right] = \sigma_0 \left\{1 - \alpha_{\mathrm{th}} \frac{E}{\sigma_0} \left[\frac{1+\nu}{1-\nu} \frac{\langle \Delta T \rangle}{2} + \frac{1}{r^2} \int_0^r r' \Delta T(r') dr'\right]\right\}. \tag{S18}$$

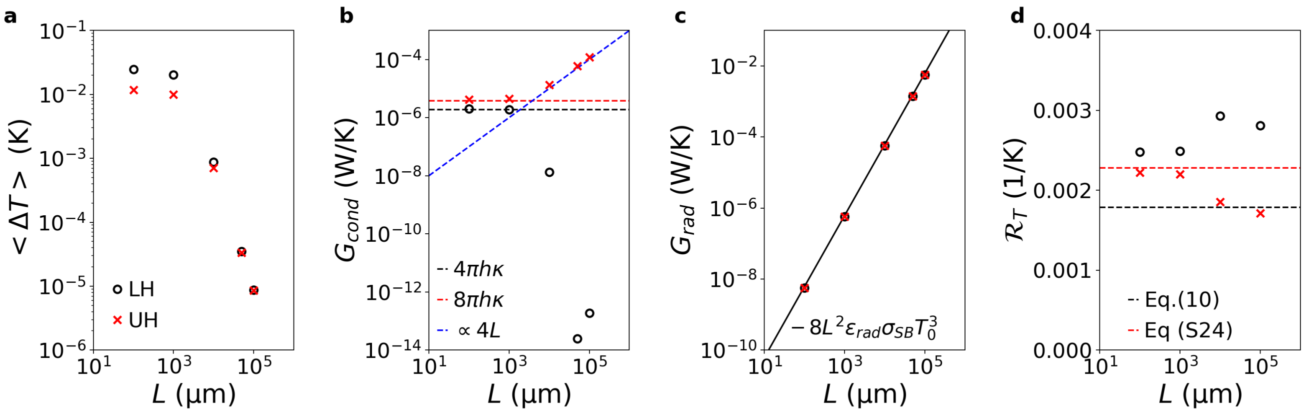

Figure S9. **Heat localization in membranes.** **a** FEM simulated mean temperature rise of square drumheads for localized (LH, black circles) and uniform heating (UH, red crosses) **b** Corresponding thermal conductance due to conduction. The blue curve shows the linear increase in G_{cond} as a function of the drumhead perimeter $4L$ for UH. **c** Corresponding thermal conductance due to radiation. **d** Corresponding relative temperature responsivity. FEM parameters: $\rho = 3000$ kg/m^3, $c_p = 700$ J/(kg K), $\kappa = 3$ W/(m $\cdot$ K), $E = 250$ GPa, $\sigma_0 = 200$ MPa, $\nu = 0.23$, $\alpha_{\mathrm{th}} = 2.2$ ppm/K, $\varepsilon_{\mathrm{rad}} = 0.05$, $\alpha_{\mathrm{abs}} = 0.5$ %, $h = 50$ nm.

Hence, this thermal stress depends on the temperature profile on the drumhead, as well as the temperature responsivity. For a uniformly distributed temperature, the integral becomes independent of r and equal to $\langle \Delta T \rangle /2$, leading to $\mathscr{R}_T$ of the form given in Eq. (10) in the main text. This is the case of large drumheads ($L > 1$ mm) under uniform heating (red crosses), as shown in Fig. S9d. For $L < 1$ mm, the temperature profile cannot be assumed constant anymore, and the temperature responsivity is given by [4]

$$\mathscr{R}_T = -\frac{\alpha_{\mathrm{th}}}{2(1-\nu)}\frac{E}{\sigma_0}[2-\nu-0.642(1-\nu)] \tag{S19}$$

It can clearly be seen that, the localization (LH, $L > 1$ mm) improves the temperature responsivity, leading to a two-fold improvement in the overall power responsivity. Overall, Eq. (10) and (S24) diverge from the temperature responsivity obtained with Eq. (S18) between $1-20$ %, from the largest to the smallest drumhead. This has been shown also in ref. [2].

C. Trampoline

For the trampoline design, only heating sources impinging onto the central pad are considered ($r \leq L/2$). Heat conduction losses are here constrained along the four tethers, each of length L_t, connecting the pad to the frame (see main text). The Fourier law at thermal equilibrium gives

$$P_0 = \frac{4wh}{L_{\mathrm{t}}}\kappa \Delta T_{\mathrm{max}} \tag{S20}$$

For an illumination only on the central pad, the β factor is constant and $\beta = 1$, as supported by FEM simulations. These have been performed for tightly focused Gaussian beam impinging in the center of the five trampolines characterized experimentally in the main text. Fig. S10 displays the corresponding temperature field along a X-cut line. It is composed of a membrane-like temperature distribution within the central pad (shadowed regions), and a linear string-like profile along the tethers. Since the temperature gradient in the central pad is smaller than the gradient at the tethers, and remains constant for different heating source positions, $\beta = 1$ (see also Fig. S11).

1. *Heat localization*

Heating localization has been also studied for trampolines. The greater (lesser) localization of the heat source does not improve (worsen) the overall relative power responsivity, as clearly shown in Fig. S11. Here, the ratio between the FEM simulated power

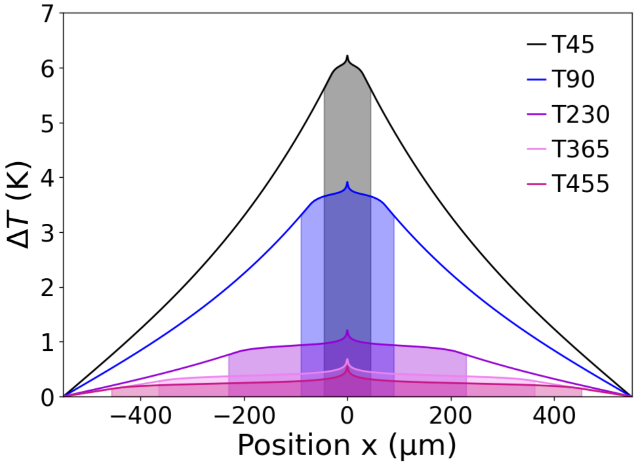

Figure S10. **Trampoline temperature profile.** FEM simulated temperature distribution along a X-cut line, for the five different trampoline dimensions analyzed experimentally. The shaded regions denote the central areas. Input optical parameters: input power $P_0 = 10\ \mu$W, beam waist $w_0 = 1\ \mu$m. FEM parameters: $\rho = 3000$ kg/m^3, $c_p = 700$ J/(kg K), $\kappa = 3$ W/(m$\cdot$K), $E = 250$ GPa, $\sigma_0 = 200$ MPa, $\nu = 0.23$, $\alpha_{\text{th}} = 2.2$ ppm/K, $\varepsilon_{\text{rad}} = 0.05$, $\alpha_{\text{abs}} = 0.5$ %, $w = 5\ \mu$m, $h = 50\ nm$.

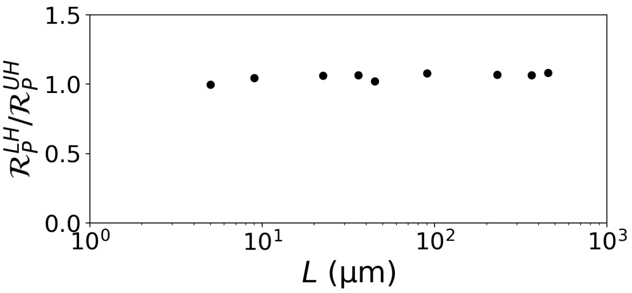

Figure S11. **Heat localization in trampolines.** Ratio between power responsivity for a localized (LH) and uniform (UH) heating condition. FEM parameters: $\rho = 3000$ kg/m^3, $c_p = 700$ J/(kg K), $\kappa = 3$ W/(m$\cdot$K), $E = 250$ GPa, $\sigma_0 = 200$ MPa, $\nu = 0.23$, $\alpha_{\text{th}} = 2.2$ ppm/K, $\varepsilon_{\text{rad}} = 0.05$, $\alpha_{\text{abs}} = 0.5$ %, $w = 5\ \mu$m, $h = 50$ nm.

responsivity in localized and uniform heating conditions is displayed, showing a value of unity for all the central pad side lengths analyzed here.

Table S3 C 1 shows a summary of the shape s_f and β factors for the different designs.

Table S1. Shape factor s_f and β factor for a square drumhead resonator with side length L.

Design	$s_f(\mathbf{r}, L, w_0)$	$\beta(\mathbf{r}, L, w_0)$
String	$\frac{4hw_{str}}{L-4\frac{\mathbf{r}^2}{L}}$	$\frac{1}{2}$
Drumhead	$\frac{4\pi h}{2cosh^{-1}\left(\frac{L^2/\pi+w_0^2-\mathbf{r}^2}{2Lw_0/\sqrt{\pi}}\right)+1}$	$\frac{1-\frac{1}{2}\left(\frac{w_0^2\pi}{L^2}\right)}{1-ln\left(\frac{w_0^2\pi}{L^2}\right)}\left(1-\frac{\mathbf{r}^2\pi}{L^2}\right)$
Trampoline	$\frac{4wh}{L_t}$ for $\lvert r\rvert < L/2$	1

S4. MECHANICS OF THE TRAMPOLINE

A trampoline resonator can be modeled using a lumped element approach, where an effective mass $m_{\rm eff}$ is connected to a fixed frame (the square window of side length $L_{\rm w}$), via a spring of constant $k_{\rm eff}$ (representing the diagonal four tethers). Under the assumption of a tensile force N applied on the unsuspended thin film of thickness h, and further considering that the resulting strain ε remains constant after the release process (since the distance between clamping points is unchanged), the balance of the tensile force can be expressed as [5]

$$\frac{N}{hE} = const = \varepsilon(x)w(x) = \frac{\sigma(x)}{E}w(x). \tag{S21}$$

$w(x)$ denotes the local width of the geometry, function of the coordinate x along a cut-line. From Eq. (S21), it can be seen that the tethers concentrate higher stress $\sigma_{\rm t}$ than the central pad, due to a reduction in cross-section. This is clearly illustrated in Fig. S12a, where a cut-line along the x coordinate is shown for FEM simulated trampolines with a Bezier profile (all simulations discussed in this section are conducted with $P_0 = 0$ μW since only the mechanical properties are analyzed). Moreover, as the tethers shorten (for high L values), this stress further increases. The FEM model includes also the chip, to better show the stress distribution. For clarity, FEM simulations have been also performed for the simplest trampoline geometry: a central square pad of area L^2 and effective mass $m_{\rm eff,c}$, connected to the frame via four tethers along its two diagonals, each of length $L_{\rm t}$ and effective mass $m_{\rm eff,t}$. For such a trampoline oscillating at its fundamental resonance frequency ω_0, the effective spring constant $k_{\rm eff}$ can be modelled as that of a string of length $L_{\rm t}$, under a prestress $\sigma_0(1-\nu)$, which is given by

$$k_{\rm eff}(\sigma_0, L_{\rm t}) = \frac{\pi^2}{2}\frac{wh}{L_{\rm t}}\sigma_0(1-\nu) \tag{S22}$$

with σ_0 denoting the nominal stress of the unstructured thin film, and with the factor $(1-\nu)$ accounting for the transverse strain relaxation upon release. From Eq. (S22), it is possible to extract the stress concentrated at the tethers

$$\sigma_{\rm t} = \frac{2}{\pi^2}\frac{1}{wh}k_{\rm eff}\frac{\sqrt{2}L_{\rm w}}{2} = \frac{\sqrt{2}L_{\rm w}}{2L_{\rm t}}\sigma_0(1-\nu). \tag{S23}$$

Hence, $\sigma_{\rm t}$ is directly proportional to the ratio of the trampoline diagonal length ($\sqrt{2}L_{\rm w}$) to the total length of the two parallel tethers ($2L_{\rm t}$). Fig. S12b displays this theoretical stress component (S23) as a function of the central pad side length L (black curve), closely aligning with the FEM results (red squares). As expected, the stress increases with L. For comparison, the FEM results for the trampolines with a Bezier profile are also displayed (black circles). Below a critical side length ($L \leq L_c \approx 500$ μm), the stress at the tethers grows faster with L than what observed for the square design. For $L > L_c$, the tethers' stress drops down, as expected from Eq. (S21). Indeed, their width increases with L in this region, conversely to the square design case, for which w is constant. This increase in w compensates for the stress reduction, making the Bezier trampolines stiffer than the square design for $L > L_c$ (Fig. S12c), consistent with the FEM simulated fundamental resonance frequency (see below, Fig. S12f). Fig. S12c clearly illustrates this compensation with the product tethers' stress-width as a function of L. Fig. S12d shows the corresponding effective spring constant $k_{\rm eff}$ (S22) as a function of L. Different power laws are displayed to illustrate the change in spring constant with central area growth. For $L^2 < 200^2$ μm^2, the stiffness matches the case of a simple string resonator, as here the trampoline is a cross-string structure. For $L^2 > 200^2$ μm^2, the stiffness increases significantly, due to stress concentration at the tethers.

From the modeshape of the trampoline's fundamental resonance, its effective mass $m_{\rm eff}$ can be written as

$$m_{\rm eff} = m_{\rm c,eff} + m_{\rm t,eff} = \rho\, h \left(L^2 + \frac{4\, w\, L_{\rm t}}{2}\right), \tag{S24}$$

with ρ denoting the resonator mass density. The tether's effective mass $m_{\rm t,eff}$ is the same as for the string, $m_{\rm s,eff} = 0.5\, m_0$. For the central pad, $m_{\rm c,eff}$, its full inertial mass is accounted for as the entire pad is being displaced for the fundamental mode. Fig. S12e displays $m_{\rm eff}$ as a function of the central pad side length L. Two regimes can be seen: for $L^2 < 100^2$ μm^2, the mass remains almost constant, as the reduction in tether length is counterbalanced by the growth of the central pad; for $L^2 > 100^2$ μm^2, the central pad fully defines the effective mass, growing here as $m_{\rm eff} \propto L^2$. It is worth noting that, in the range 100 μm $< L <$ 500 μm, the effective mass grows faster than the spring constant, reducing the overall resonance frequency in this region (Fig. S12f). Fig. S12f compares the FEM results for square and Bezier trampolines with the theoretical predictions for the fundamental resonance frequency $\omega_0 = \sqrt{k_{\rm eff}/m_{\rm eff}}$, showing excellent agreement. ω_0 increases faster with L for the Bezier design, due to an overall increase in tethers' stiffness, as shonw above.

Having a closed expression for the trampoline's fundamental resonance frequency allows for the extraction the relative temperature responsivity $\mathscr{R}_T$. Whenever the resonator experiences a mean temperature rise $\langle \Delta T \rangle$, the effective stiffness is reduced

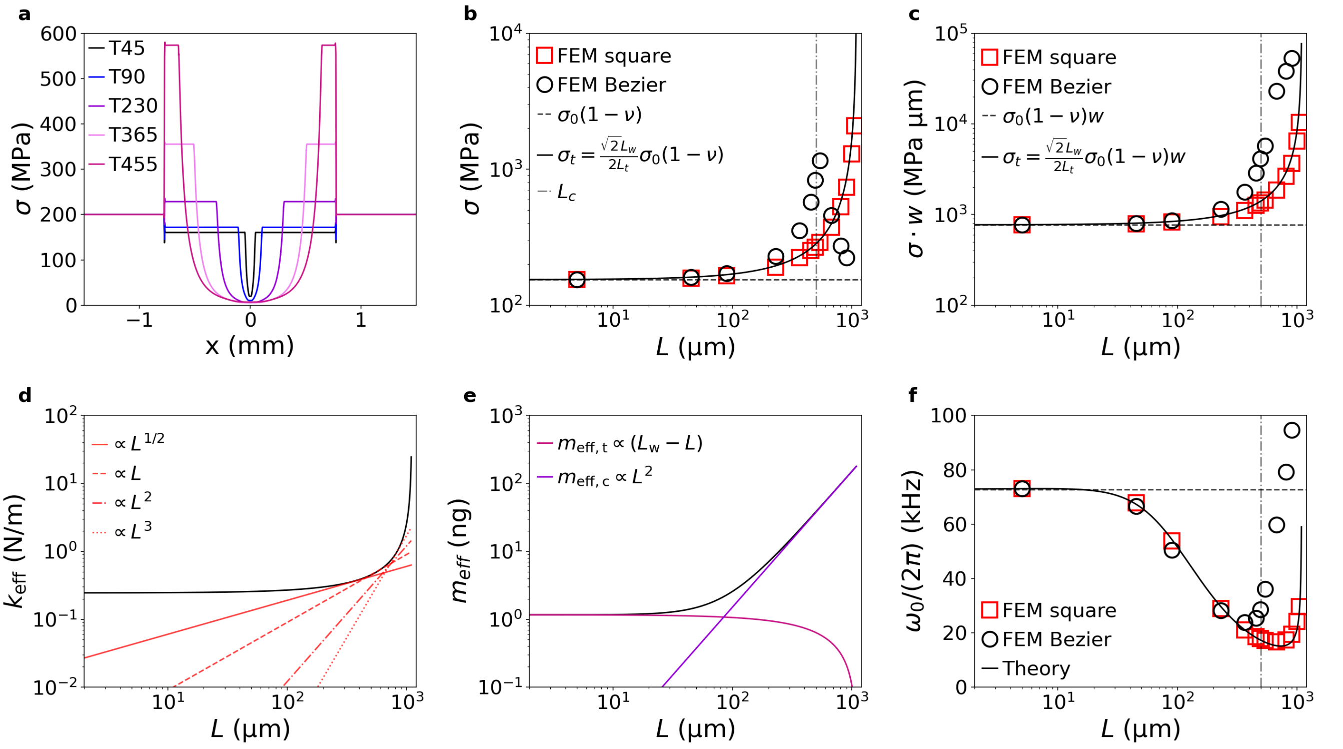

Figure S12. **Mechanics of the trampoline. a** X-cut stress profile in trampolines with a central pad with a Bezier curve profile. **b** Tethers stress as a function of the central pad side length L. Red squares: FEM simulations for a trampoline with a square design of the central pad. Black circles: FEM simulations for a trampoline with a Bezier profile design for the pad. Black curve: theory (S23). Vertical dashed dotted line: critical central pad side length L_c. For $L > L_c$, the tether width at the clamping points for a Bezier trampoline increases with L, changing the boundary conditions relative to the square design. **c** Product tether's stress-width $\sigma_t \cdot w$ as a function of L. For $L > L_c$, the reduction in stress is compensated by the increase in width at the clamping points. **d** Square trampoline spring constant (S22) as a function of L. Displayed are also different power laws L^ζ for clarity. **d** Trampoline effective mass as a function of L. Pink curve: tethers' effective mass. Purple: central pad's effective mass. **f** Fundamental resonance frequency as a function of L. FEM and model parameters: $\rho = 3000$ kg/m^3, $E = 250$ GPa, $\sigma_0 = 200$ MPa, $\nu = 0.23$, $h = 50$ nm. For the square design, $w = 5$ μm always; for the Bezier design, $w = 5$ μm for $L \leq L_c$.

by the built-in thermal stress along the tethers

$$k_{\text{eff}}(T) = \frac{\pi^2}{2}\frac{wh}{L_{\text{t}}}(1-\nu)\left[\sigma_0 - \alpha_{\text{th}}E\langle\Delta T\rangle\right] = k_{\text{eff}}(\sigma_0, L_{\text{t}})\left[1 - \frac{\alpha_{\text{th}}E}{\sigma_0}\langle\Delta T\rangle\right]. \tag{S25}$$

Substituing Eq. (S25) into the resonance frequency equation gives

$$\omega_0(T) = \sqrt{\frac{k_{\text{eff}}(T)}{m_{\text{eff}}}} = \sqrt{\frac{k_{\text{eff}}(\sigma_0, L_{\text{t}})}{m_{\text{eff}}}}\sqrt{1 - \frac{\alpha_{\text{th}}E}{\sigma_0}\langle\Delta T\rangle} \approx \omega_0(0)\left[1 - \frac{\alpha_{\text{th}}E}{2\sigma_0}\langle\Delta T\rangle\right] \tag{S26}$$

Therefore, the temperature responsivity $\mathscr{R}_T$ for a trampoline is recovered (see Eq. 12 in the main text).

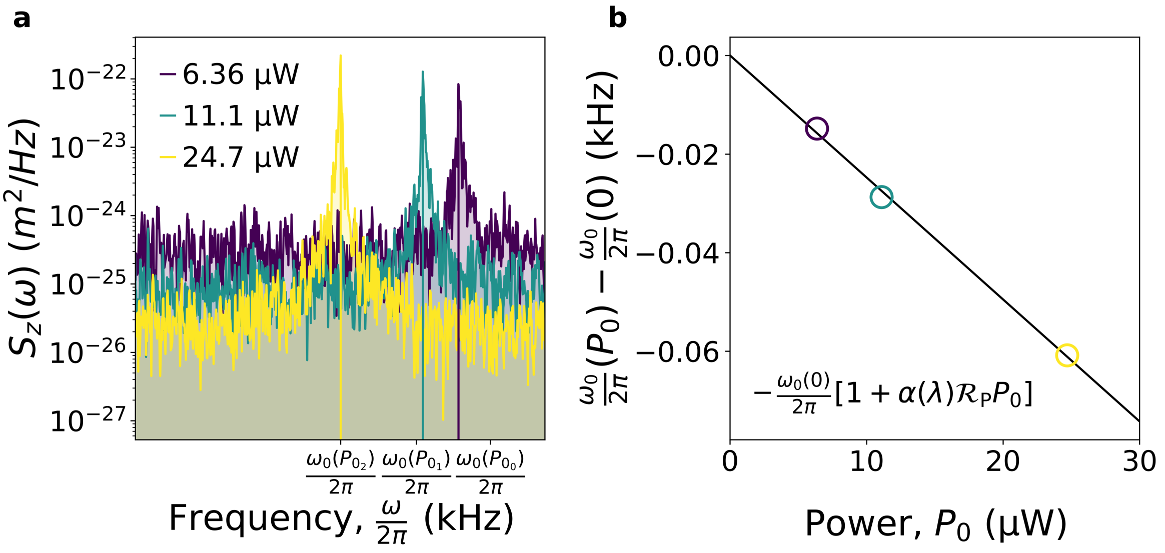

Figure S13. **Power responsivity characterization. a** Displacement power spectral density $S_z(\omega)$ of the fundamental flexural mode, for varying input power laser P_0. **b** The extracted resonance frequency $\omega_0/2\pi$ as a function of the input power P_0. From the fitting (black curve), the power responsivity is extracted.

S5. MEASUREMENT PROCEDURE

A. Relative power responsivity $\mathscr{R}_\mathrm{P}$

The relative power responsivity $\mathscr{R}_\mathrm{P}$ is evaluated experimentally from the frequency detuning of the undriven displacement power spectral density $S_z(\omega)$ for varying input laser power P_0, as shown in Fig. S13a for the fundamental resonance frequency of a drumhead resonator. For each input power, the resonance frequency is extracted from the spectrum and plotted as a function of P_0, as shown in Fig. S13b. The data are fitted with Eq. (S1), where the slope is directly proportional to the power responsivity $\mathscr{R}_\mathrm{P}$ [6, 7].

B. Thermal time constant τ_th

The thermal time constant of each resonator has been characterized experimentally with the 90-10 method [8, 9]. In the case of PLL tracking, the resonance frequency $\omega_0/2\pi$ was first phase-locked with a measurement bandwidth $f_\mathrm{PLL} \geq 500$ Hz, ensuring that the response time of the resonator was not limited by the experimental set-up. Once phase-locked, the resonance frequency was detuned by varying the optical input power P_0 of the interferometric laser (HeNe, 633 nm), as shown in Fig. S14 (right). For a single optical power step (Fig.S14, left), the resonance frequency shifted by an amount $\overline{\Delta\omega_0}/2\pi$. The 90-10 method was then applied to determine the fall time $\tau_{90} - \tau_{10}$ of the resonator. From these data, the first-order low-pass time constant τ_th (see Eq. (3) in the manuscript) was calculated as follows

$$\tau_\mathrm{th} = \frac{\tau_{90} - \tau_{10}}{\ln(9)}. \tag{S27}$$

C. Frequency noise

1. PLL settings

The resonance frequency of the fundamental flexural out-of-plane mode of each resonator is tracked in time with PLL or SSO. The transfer functions of the thermomechanical and detection phase noise for the SSO are given in the main text in Eq. 33. For

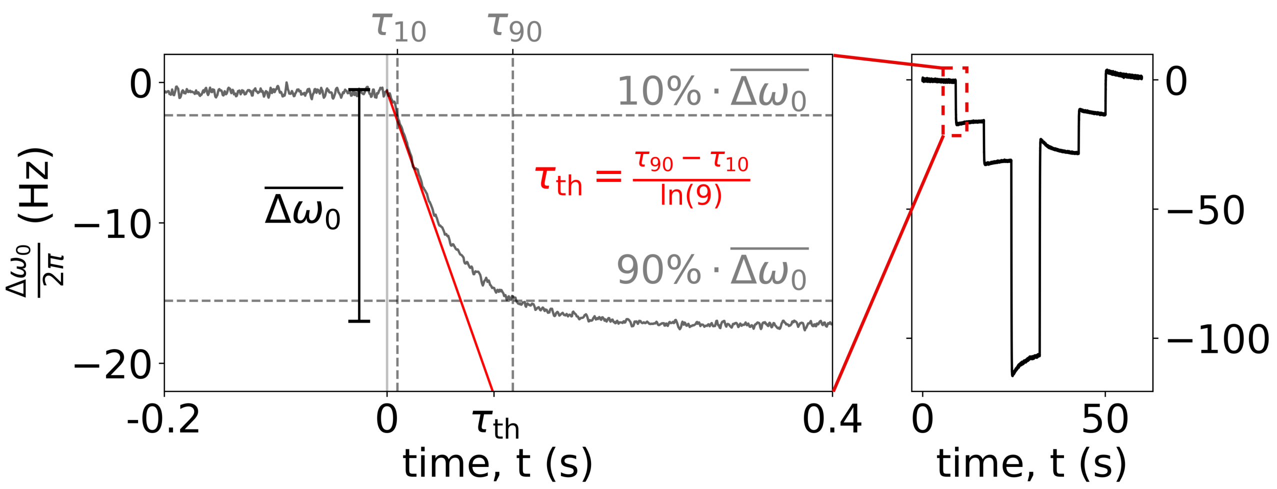

Figure S14. **Thermal time constant characterization.** Resonance frequency $\omega_0/2\pi$ as a function of time for a step increase in optical power.

the PLL scheme the two transfer functions are given by [10]

$$H^{\rm PLL}_{\theta_{\rm thm}}(i\omega) = \frac{1}{\tau_{\rm mech}} \frac{(i\omega k_P + k_I)H_{\rm L}(i\omega)}{-\omega^2 + i\frac{\omega}{\tau_{\rm mech}} + (i\omega k_P + k_I)H_{\rm L}(i\omega)} \tag{S28}$$

$$H^{\rm PLL}_{\theta_{\rm det}}(i\omega) = \frac{1}{\tau_{\rm mech}} \frac{1}{H_{\rm mech}(i\omega)} \frac{(i\omega k_P + k_I)H_{\rm L}(i\omega)}{-\omega^2 + i\frac{\omega}{\tau_{\rm mech}} + (i\omega k_P + k_I)H_{\rm L}(i\omega)}, \tag{S29}$$

with $k_{\rm P}$ and $k_{\rm I}$ being the proportional and integral coefficients, respectively. They are chosen to be [10]

$$k_{\rm P} = 2\pi f_{\rm PLL} = \frac{1}{\tau_{\rm PLL}}, \tag{S30}$$

$$k_{\rm I} = \frac{k_{\rm P}}{\tau_{\rm mech}}, \tag{S31}$$

with $f_{\rm PLL}$ denoting the PLL loop bandwidth.

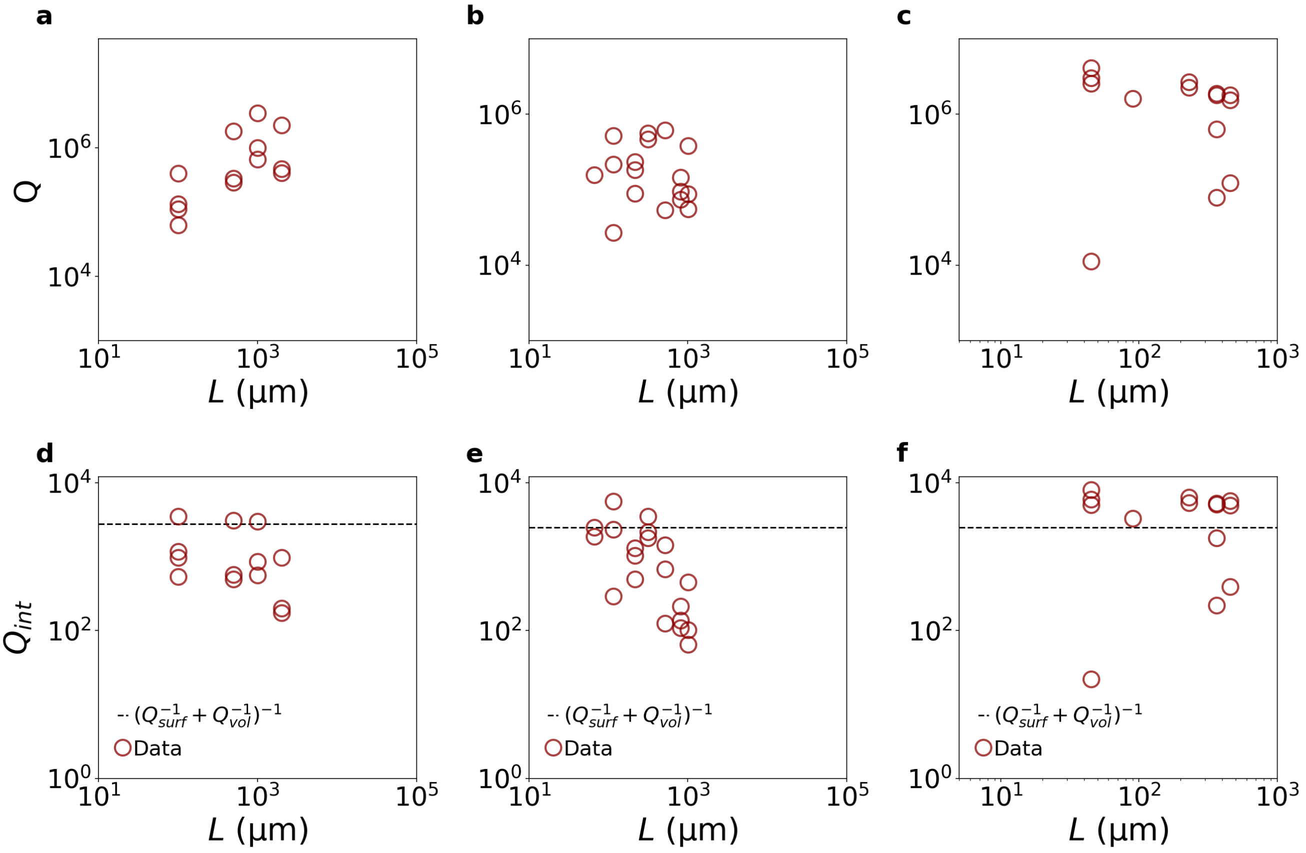

Figure S15. **Q factors.** Measured Q factor for: **a** strings, **b** drumheads, **c** trampolines. **d**-**f** Corresponding intrinsic Q.

2. *Experimental Q factors*

The quality factor of each resonator analyzed in the main text has been measured for the theoretical calculations of the AD, due to the Q-dependence of the additive phase noise (Eq. 27 in the main text). Q are measured with the ring-down method [1]. Fig. S15 displays the experimental values for the damping diluted Q and intrinsic Q_{int}. The latter results to be mainly dominated by surface losses Q_{surf}, as expected for this thickness, for all the analyzed structures [11]. Moreover, chip mounting constitute here another source of mechanical dissipation, as observed in the data scattering [12].

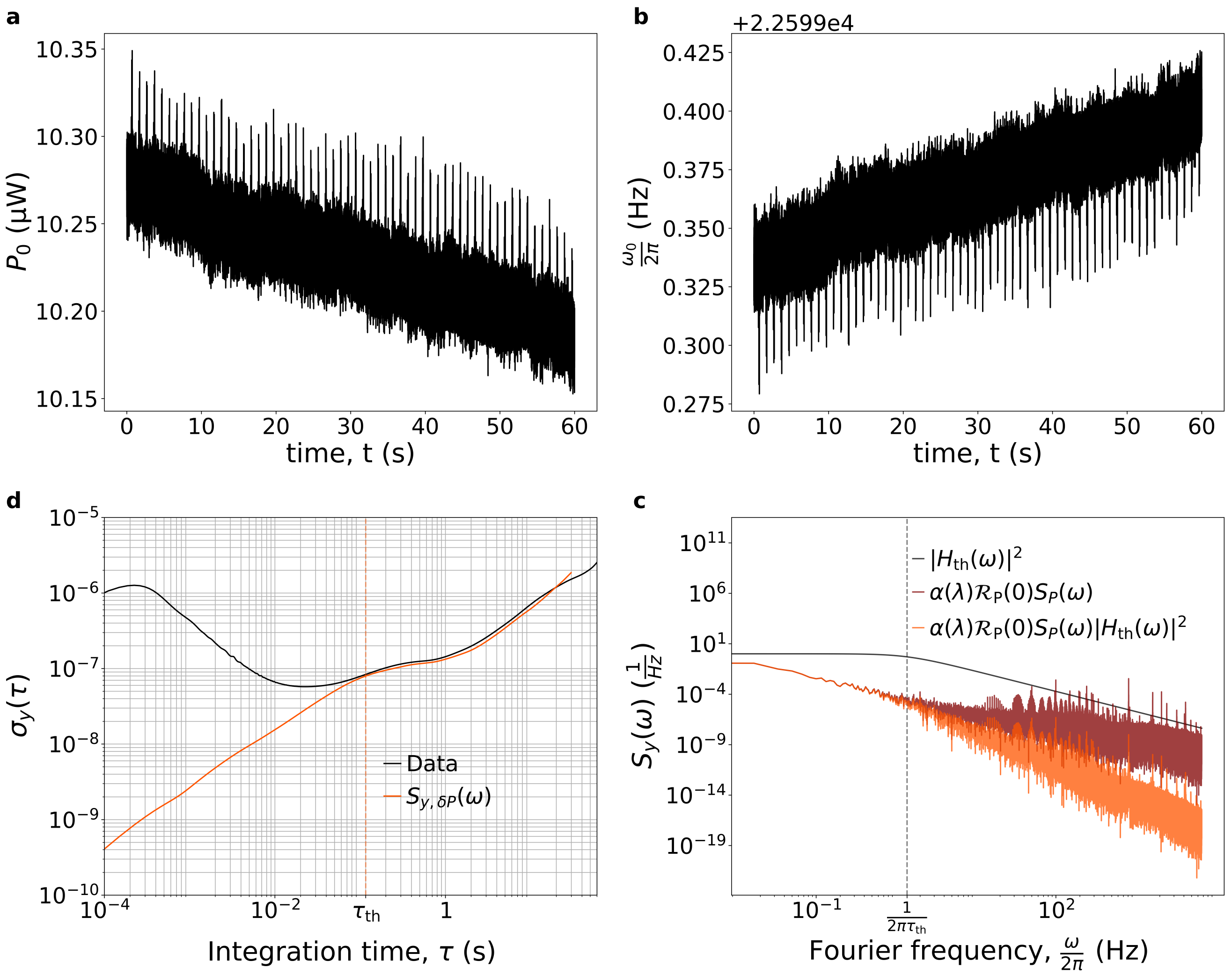

Figure S16. **Photothermal back-action.** **a** Recorded power signal. **b** Corresponding frequency signal obtained using Eq. (S1). **c** Fractional frequency PSD before (dark red) and after (orange) low-pass filtering, of transfer function (black solid curve).

3. Photothermal back-action

To understand the magnitude of the photothermal back-action on the final fractional frequency fluctuations of the resonator, the optical power PSD $S_P(\omega,\lambda)$ has been measured recording the optical power in time $P_0(t)$ for 2 minutes, as shown in Fig. S16a. This power is then converted in frequency through the use of Eq. (S1), as shown in Fig. S16b.
The resulting frequency signal is then filtered with a low-pass filter with transfer function $H_{\text{th}}(\omega) = (1 + i\omega\tau_{\text{th}})^{-1}$, to account for the finite time response of the resonator. Fig. S16c shows the fractional frequency PSD of the signal before (dark red) and after (orange) the filtering. The Allan deviation of the filtered signal is then computed using Eq. (29) in the manuscript, as shown in Fig. S16.

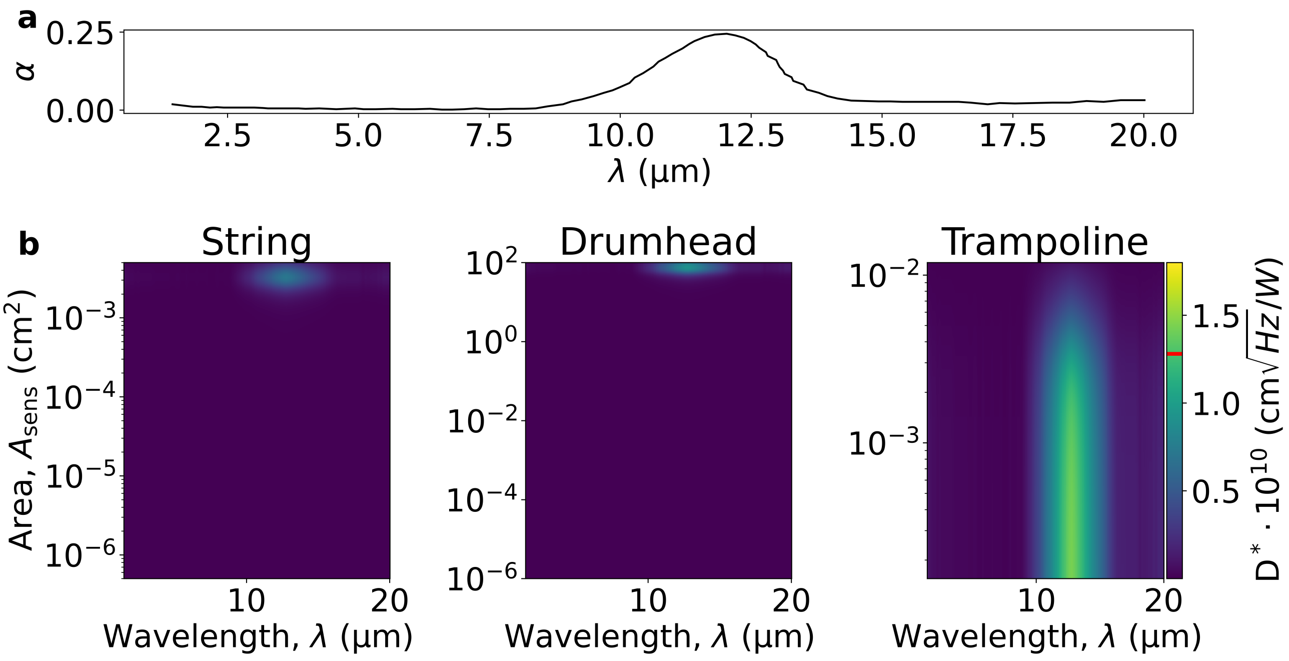

Figure S17. **Specific detectivity.** **a** Absorption spectrum taken from [15]. **b** Specific detectivity D^* as a function of the wavelength and resonator's area.

S6. SPECIFIC DETECTIVITY

The specific detectivity $D^* \equiv \sqrt{A_{\text{sens}}}/\text{NEP}$ is used as main parameter to measure the performance of IR detectors [13]. In the regime where temperature fluctuations dominate the frequency noise of the sensor, where $S_y(\omega) \approx S_{y_{\text{th}}}(\omega)$, the NEP can be written as [14]

$$\text{NEP} \approx \text{NEP}_{\text{th}} = \frac{\sqrt{4A_{\text{sens}}\varepsilon_{\text{rad}}\sigma_{\text{SB}}k_{\text{B}}T_0^5}}{\alpha(\lambda)}. \tag{S32}$$

Hence, in this regime, the specific detectivity for a single side coupled to the environment reaches its maximum and becomes independent of the area

$$D^* = \frac{\sqrt{A_{\text{sens}}}}{\text{NEP}_{\text{th}}} = \alpha(\lambda)\sqrt{\frac{1}{16\varepsilon_{\text{rad}}\sigma_{\text{SB}}k_{\text{B}}T_0^5}}. \tag{S33}$$

For a black-body, $\alpha(\lambda) = \varepsilon_{\text{rad}} = 1$, $D^* \approx 1.8 \cdot 10^{10}$ cm$\sqrt{\text{Hz}}$/W [14]. In the case where both side are coupled to the environment, but only one is absorbing light, $D^* \approx 1.28 \cdot 10^{10}$ cm$\sqrt{\text{Hz}}$/W (red line in the scale bar in Fig. S17).

Fig. S17 shows the calculations of D^* as a function of the resonators' area and excitation wavelength, in the ideal case where the photothermal back-action is negligible. For all designs, a region of maximum detectivity is found, where D^* is independent of the area A_{sens}, aligning the resonators' performance. However, drumheads and trampolines offer the advantage of less stringent optical requirements, needing less precise focusing of the incoming IR light.

[1] S. Schmid, L. G. Villanueva, and M. L. Roukes, *Fundamentals of Nanomechanical Resonators* (Springer International Publishing, 2023).
[2] C. Zhang, M. Giroux, T. A. Nour, and R. St-Gelais, Phys. Rev. Appl. **14**, 024072 (2020).
[3] T. L. Bergman, A. Lavine, and F. P. Incropera, *Fundamentals of heat and mass transfer.* (John Wiley, 2017) p. 966.
[4] M. Kurek, M. Carnoy, P. E. Larsen, L. H. Nielsen, O. Hansen, T. Rades, S. Schmid, and A. Boisen, Angew. Chem., Int. Ed. **56**, 3901 (2017).
[5] S. A. Fedorov, N. J. Engelsen, A. H. Ghadimi, M. J. Bereyhi, R. Schilling, D. J. Wilson, and T. J. Kippenberg, Physical Review B **99**, 10.1103/PhysRevB.99.054107 (2019).
[6] M. H. Chien, M. Brameshuber, B. K. Rossboth, G. J. Schütz, and S. Schmid, Proc. Natl. Acad. Sci. U.S.A **115**, 11150 (2018).
[7] P. Sadeghi, M. Tanzer, N. Luhmann, M. Piller, M. H. Chien, and S. Schmid, Phys. Rev. Appl. **14**, 024068 (2020).
[8] L. Duraffourg, L. Laurent, J. S. Moulet, J. Arcamone, and J. J. Yon, Micromachines **9**, 10.3390/mi9080401 (2018).
[9] M. Piller, J. Hiesberger, E. Wistrela, P. Martini, N. Luhmann, and S. Schmid, IEEE Sens. J. **23**, 1066 (2023).
[10] A. Demir, J. Appl. Phys. **129**, 044503 (2021).
[11] L. G. Villanueva and S. Schmid, Phys. Rev. Lett. **113**, 227201 (2014).
[12] S. Schmid, K. D. Jensen, K. H. Nielsen, and A. Boisen, Physical Review B - Condensed Matter and Materials Physics **84**, 10.1103/PhysRevB.84.165307 (2011).
[13] A. Rogalski, *Infrared and terahertz detectors* (CRC press, 2019).
[14] N. Snell, C. Zhang, G. Mu, A. Bouchard, and R. St-Gelais, Phys. Rev. Appl. **17**, 044019 (2022).
[15] M. Piller, P. Sadeghi, R. G. West, N. Luhmann, P. Martini, O. Hansen, and S. Schmid, Appl. Phys. Lett. **117**, 034101 (2020).
